# Raman spectroscopy online monitoring of biomass production, intracellular metabolites and carbon substrates during submerged fermentation of oleaginous and carotenogenic microorganisms

**DOI:** 10.1186/s12934-023-02268-y

**Published:** 2023-12-18

**Authors:** Simona Dzurendova, Pernille Margrethe Olsen, Dana Byrtusová, Valeria Tafintseva, Volha Shapaval, Svein Jarle Horn, Achim Kohler, Martin Szotkowski, Ivana Marova, Boris Zimmermann

**Affiliations:** 1 Faculty of Science and Technology, Norwegian University of Life Sciences, Drøbakveien 31, P.O. Box 5003, 1432 Ås, Norway; 2 Faculty of Chemistry, Biotechnology and Food Science, Norwegian University of Life Sciences, P.O. Box 5003, 1432 Ås, Norway; 3 Institute of Food Science and Biotechnology, Faculty of Chemistry, Brno University of Technology, Purkyňova 464/118, Brno, 61200 Czech Republic

**Keywords:** Process analytical technology, Raman spectroscopy, Infrared spectroscopy, Real-time monitoring, Partial least squares (PLS) regression, Lipids, Carotenoids, *Rhodotorula*, *Schizochytrium*

## Abstract

**Background:**

Monitoring and control of both growth media and microbial biomass is extremely important for the development of economical bioprocesses. Unfortunately, process monitoring is still dependent on a limited number of standard parameters (pH, temperature, gasses etc.), while the critical process parameters, such as biomass, product and substrate concentrations, are rarely assessable in-line. Bioprocess optimization and monitoring will greatly benefit from advanced spectroscopy-based sensors that enable real-time monitoring and control. Here, Fourier transform (FT) Raman spectroscopy measurement via flow cell in a recirculatory loop, in combination with predictive data modeling, was assessed as a fast, low-cost, and highly sensitive process analytical technology (PAT) system for online monitoring of critical process parameters. To show the general applicability of the method, submerged fermentation was monitored using two different oleaginous and carotenogenic microorganisms grown on two different carbon substrates: glucose fermentation by yeast *Rhodotorula toruloides* and glycerol fermentation by marine thraustochytrid *Schizochytrium* sp. Additionally, the online FT-Raman spectroscopy approach was compared with two at-line spectroscopic methods, namely FT-Raman and FT-infrared spectroscopies in high throughput screening (HTS) setups.

**Results:**

The system can provide real-time concentration data on carbon substrate (glucose and glycerol) utilization, and production of biomass, carotenoid pigments, and lipids (triglycerides and free fatty acids). Robust multivariate regression models were developed and showed high level of correlation between the online FT-Raman spectral data and reference measurements, with coefficients of determination (R^2^) in the 0.94–0.99 and 0.89–0.99 range for all concentration parameters of *Rhodotorula* and *Schizochytrium* fermentation, respectively. The online FT-Raman spectroscopy approach was superior to the at-line methods since the obtained information was more comprehensive, timely and provided more precise concentration profiles.

**Conclusions:**

The FT-Raman spectroscopy system with a flow measurement cell in a recirculatory loop, in combination with prediction models, can simultaneously provide real-time concentration data on carbon substrate utilization, and production of biomass, carotenoid pigments, and lipids. This data enables monitoring of dynamic behaviour of oleaginous and carotenogenic microorganisms, and thus can provide critical process parameters for process optimization and control. Overall, this study demonstrated the feasibility of using FT-Raman spectroscopy for online monitoring of fermentation processes.

**Supplementary Information:**

The online version contains supplementary material available at 10.1186/s12934-023-02268-y.

## Introduction

Microbial biotechnology is a rapidly growing field since fermentation bioprocesses are considered more sustainable and, in a number of cases, more efficient than traditional chemical processes. Nowadays, heterotrophic bioreactor fermentations are used to produce a wide range of products, such as food, biofuels, enzymes, antibiotics, and various platform chemicals and fine chemicals. For example, under nitrogen starvation conditions, oleaginous and carotenogenic microorganisms can accumulate significant amounts of lipids in the form of triacylglycerols (triglycerides, TAGs), free fatty acids (FFAs) and carotenoids in intracellular lipid bodies [1, 2]. Moreover, they can metabolize different types of substrates, from five- and six-carbon sugars and glycerol, to fat residues, thus lowering the cost of fermentation via utilization of waste substrates [3, 4].

Process monitoring and control are important aspects of microbial fermentation, as they are essential for improving the process stability and ensuring the quantity and quality of the final product [5]. In this regard, online monitoring with real-time data on fermentation parameters enables the development of more robust process models for prediction, optimization, and control of fermentation. While real-time data for several process parameters, including pH, temperature, and oxygen levels, is readily available, some crucial parameters, like substrate and product concentrations, are rarely measurable in real-time. Traditional methods of monitoring substrate and product concentration involve withdrawing samples from bioreactors and analyzing them offline by costly and time-consuming chromatographies or assay kits, which can lead to a delay in obtaining results and making appropriate adjustments to the fermentation process [5].

In the last decade, Raman spectroscopy-based sensors started to emerge as powerful process analytical technology (PAT) monitoring systems to provide real-time data for fermentation processes [6–9]. Raman spectroscopy is considered a fast, low-cost, and highly sensitive vibrational spectroscopy technique for non-destructive analysis of bioprocess substrates and products. It is an excellent technique for obtaining comprehensive and detailed information in bioreactor fermentations, as it can simultaneously detect various chemical constitutes present in the bioprocess via characteristic functional groups. Online Raman spectroscopy monitoring can be conducted not only by using an immersion probe in a bioreactor [10, 11], but also in a non-invasive way, by using a flow cell in recirculatory loop [12], or by measuring through a transparent bioreactor window [7]. Such non-invasive monitoring approaches have great advantage since they allow using simpler, cheaper, and more versatile sensors, while simultaneously greatly reducing the risk of contamination which is a pervasive problem in fermentation processes. Raman spectra of microbial biomass exhibit a wide range of signals associated with different cellular constituents, including lipids, proteins, pigments, and carbohydrates [13]. Moreover, Raman spectra of growth media can be used to measure the concentration of nutrients and extracellular metabolites, such as monosaccharides, alcohols, and organic acids [10, 14]. Raman spectral datasets can be large, complex and extremely information-rich, and therefore chemometrics, conventional machine learning, and deep learning methods are often used for analysis, interpretation and prediction.

Raman scattering intensities are relatively weak, making it difficult to detect analytes present at low concentrations in a fermentation broth. This problem is exacerbated by the presence of fluorescence interference, which is a common occurrence in Raman spectroscopy of biological samples. Use of near-infrared (NIR) excitation lasers, such as those at 993 and 1064 nm, can reduce fluorescence interference problems in Raman spectroscopy [15]. Unfortunately, compared to ultraviolet and visible lasers, NIR excitation lasers result with substantially lower Raman intensity. Thus, they require dedicated Fourier transform (FT) Raman spectrometers with interferometer and FT processor for signal-to-noise enhancement. In the last decade, FT-Raman spectroscopy has gained momentum in analyses of biological samples [9, 16]. At the turn of the century, FT-Raman spectroscopy has shown its potential as a PAT in biotechnology for monitoring ethanol production in yeast fermentation of glucose [17]. However, it still remains largely unexplored in the monitoring of fermentation processes.

In a previous study, it was showed that FT-Raman spectroscopy can be used in an at-line setting for simultaneous assessment of different microbial intercellular metabolites, such as lipids, carotenoids and phosphorus [13]. In the present study, the potential of FT-Raman spectroscopy for real-time online monitoring of critical fermentation process parameters was assessed, specifically biomass, intracellular metabolites and carbon substrates. The online FT-Raman spectroscopy measurements were conducted via non-invasive spectral acquisition through the steam sterilised glass flow measurement cell in a recirculatory loop, thus preserving the sterile barrier of the bioreactor. Additionally, the online FT-Raman spectroscopy approach was compared with two at-line spectroscopic methods, namely FT-Raman and FT-infrared (FTIR) spectroscopies in high throughput screening (HTS) setups. These two spectroscopy methods have shown great potential for monitoring of microbial biomass and growth media [13, 18–24]. To show the general applicability of the method, submerged fermentation was monitored using two different oleaginous and carotenogenic microorganisms grown on two different carbon substrates: glucose fermentation by the yeast *Rhodotorula toruloides* and glycerol fermentation by thraustochytrid *Schizochytrium* sp. To our knowledge, this is the first time that concentrations of several intracellular metabolites (TAGs, FFAs and carotenoid pigments), in addition to biomass production and carbon substrate utilisation, have been simultaneously monitored in a real-time setting by a single non-invasive online sensor. Moreover, this is the first study where thraustochytrid fermentation was monitored by a vibrational spectroscopy PAT system.

## Material and methods

### Microbial strains

*Rhodotorula toruloides* CCY 62-2-4 was obtained from the Culture Collection of Yeasts (Bratislava, Slovakia). *Schizochytrium* sp. ATCC 20888 was obtained from the American Type Culture Collection (Manassas, VA, USA).

### Preparation of inoculum and seed culture

The *Rhodotorula toruloides* culture was firstly refreshed from cryopreserved stocks performing agar cultivation in 2 bioreplicates. The agar medium consisted of: yeast extract 10 g/L, peptone 20 g/L, glucose 20 g/L and agar 20 g/L. The agar plates were incubated statically at 22 °C for 72 h. After the agar plate cultivation, pre-inoculum was cultivated by transferring one 10 μL loop of biomass from agar in 250 mL Erlenmeyer flask with 50 mL medium. 10% (v/v) of pre-inoculum was used for inoculum cultivation in 500 mL Erlenmeyer flask with 170 mL medium. The pre-inoculum and inoculum media contained: glucose 20 g/L, yeast extract 10 g/L and peptone 20 g/L. The inoculums were incubated at 22 °C under the constant shaking regime (140 revolutions per minute (rpm), 1.9 cm) for 24 h in 2 bioreplicates.

The *Schizochytrium* sp. culture was firstly refreshed from a cryopreserved stock (in 20% (v/v) glycerol, kept at − 80 °C). 10% (v/v) thawed culture was inoculated in 5 mL ATCC By^+^ medium consisted of: yeast extract 1 g/L, peptone 1 g/L, glucose 5 g/L, and sea salts 35 g/L, and incubated statically at 21 °C for 96 h. The seed culture was prepared by inoculating 5% (v/v) of the static preculture in 500 mL baffled shake flask with 100 mL medium consisted of: yeast extract 5 g/L, peptone 5 g/L, glycerol 30 g/L, and sea salts 35 g/L. As a general precaution against bacterial contamination, antibiotics (0.3 g/L Penicillium G and 0.3 g/L streptomycin) were added to inoculums. The inoculums were incubated at 25 °C under the constant shaking regime (170 rpm) for 48 h in 2 bioreplicates.

### Bioreactor cultivation

The bioreactor cultivation in the production media was performed in two bioreplicates in 3.0 L glass bioreactors (Infors Minifors 2, Bottmingen, Switzerland), with a working volume of 1.7 L (for *Rhodotorula* cultivation) or 2.0 L (for *Schizochytrium* cultivation). Foaming was controlled by a built-in foam sensor that regulated addition of antifoam solution. Dissolved oxygen (DO) was monitored by a pO_2_-sensor (Hamilton, Bonaduz, Switzerland) and controlled by aeration, either by using spargers with a constant airflow of 0.5 volume of air under standard conditions per volume of liquid per minute (VVM) (for *Schizochytrium* cultivation) or by changing the stirring rpm (for *Rhodotorula* cultivation). The pH was monitored by a pH-probe (Hamilton, Bonaduz, Switzerland) and controlled through automatic addition of acidic and basic solutions. The abovementioned online data was recorded by the eve bioprocess control software (Infors, Bottmingen, Switzerland). For the online spectroscopy monitoring, the bioreactor was connected to the FT-Raman spectrometer via a recirculatory loop. A Masterflex L/S peristaltic pump with convex roller design Cytoflow 3-roller pump head (both Cole-Parmer GmbH, Germany) circulated the fermentation cell suspension with 1 L/h flow rate from the bioreactor to a flow cell made of borosilicate glass for FT-Raman spectroscopy measurement, before returning the suspension to the bioreactor. A control bioreactor fermentation without the loop was also carried out.

For *Rhodotorula*, the composition of the cultivation media was the following (in g/L): glucose 52.2, yeast extract 2, KH_2_PO_4_ 4, MgSO_4_·7H_2_O 0.7. The bioreactors containing cultivation media components, except yeast extract, were autoclaved for 20 min at 121 °C. For the bioreactor containing the flow measurement cell in a recirculatory loop, the whole system (comprising the bioreactor and the bypass loop) was autoclaved. Yeast extract was autoclaved separately and injected into the bioreactors prior to inoculation. The volume of inoculum that corresponded to 10% v/v of production medium cultivation (170 mL) was up-concentrated in 50 mL cell suspension and was injected in the bioreactor. The pH was maintained at 6.5 through automatic additions of 5 M NaOH and 2 M H_2_SO_4_. The level of dissolved oxygen was kept above 30%, and the cultivation was carried out at 22 °C. Glanapon 2000 (Bussetti, Vienna, Austria) was used as antifoam. The stirring was controlled within the range of 400–800 rpm using Rushton impellers. The duration of bioreactor cultivation was 120 h with the following sampling timepoints: 4, 8, 12, 16, 20, 24, 32, 40, 48, 72, 96 and 120 h. 20–30 mL of sample was withdrawn for each timepoint to secure sufficient amount of biomass for the reference analyses.

For *Schizochytrium*, the composition of the cultivation media was the following (in g/L): glycerol 55, yeast extract 3, peptone 3, and sea salts 35. First, the bioreactors containing cultivation media components, except glycerol, were autoclaved for 20 min at 121 °C. Second, 0.3 g/L Penicillium G and 0.3 g/L streptomycin were sterilized by filtration, and added into the bioreactors as a general precaution against bacterial contamination. Finally, autoclaved glycerol was added aseptically just before inoculation of the bioreactors with 10% v/v seed culture. The pH was maintained at 7.0 through automatic additions of 5 M NaOH and 2 M HCl. The level of dissolved oxygen was kept at 20% during the first 48 h, and then reduced to 5% until the end of the cultivation, and the cultivation was carried out at 28 °C. Clerol antifoam (PMC Ouvrie, Carvin, France) was used to control foaming. The stirring was set within the range of 300–600 rpm using Rushton impellers. The duration of bioreactor cultivation was 120 h with the following sampling timepoints: 8, 16, 24, 30, 36, 42, 48, 60, 72, 84, 96 and 120 h. 50 mL of sample was withdrawn for each timepoint to secure sufficient amount of biomass for the reference analyses.

### Biomass and supernatant

The collected culture broths of *Rhodotorula* fermentations were centrifuged at 3200*g* for 5 min at 15 °C. The supernatants were stored at − 20 °C until further analytics (glucose analysis). The biomass was washed three times using distilled water, frozen at − 80 °C, followed by freeze-drying for a minimum of 48 h in FreeZone 2.5 freeze-dryer (Labconco, USA) at − 50 °C and 0.01 mbar. The collected culture broths of *Schizochytrium* sp. fermentations were centrifuged at 8000*g* for 15 min at 4 °C, and the supernatants were collected and stored at − 20 °C until further analytics (glycerol analysis). The biomass pellet was washed once with a 0.9% saline solution, frozen at − 80 °C, followed by freeze-drying in a Heto Drywinner DW6 freeze-dryer (Heto-Holten, Allerød, Denmark) at − 80 °C and < 0.1 mbar vacuum for a minimum of 24 h. The dried biomasses were used for cell dry weight (CDW) estimation, HTS-FTIR and HTS-FT-Raman vibrational spectroscopy analyses, lipid analysis, and carotenoid analysis. *Schizochytrium* supernatant was used for glycerol estimation by HPLC analysis, while *Rhodotorula* supernatant was used for glucose estimation by assay kit.

### Vibrational spectroscopy analyses

*On-line FT-Raman spectra* were recorded (for the bioreplicates in the bioreactor with the recirculatory loop) in backscattering geometry using a MultiRAM FT-Raman spectrometer (Bruker Optik GmbH, Germany) equipped with a neodymium-doped yttrium aluminium garnet (Nd:YAG) laser (1064 nm, 9394 cm^−1^), and germanium detector cooled with liquid nitrogen. The spectra were recorded every 20 min, with a total of 1024 scans, using Norton-Beer medium (*Rhodotorula* fermentation) or Blackman-Harris 4-term (*Schizochytrium* fermentation) apodization, employing a spectral resolution of 8 cm^−1^, with a digital resolution of 1.928 in *Rhodotorula* fermentation and of 3.856 cm^−1^ in *Schizochytrium* fermentation, over the range of 3785–45 cm^−1^, at 1000 mW laser power. The acquisition of 1024 scans (i.e. the acquisition time per spectrum) required approx. 15 min. Due to a software error, spectra were not acquired between 6100 and 6280 min during *Rhodotorula* fermentation. In total, 349 and 362 spectra were recorded during the *Rhodotorula* fermentation and *Schizochytrium* fermentation, respectively. The OPUS software (Bruker Optik GmbH, Germany) was used for data acquisition and instrument control.

*At-line HTS-FT-R*aman and *HTS-FTIR* spectra were recorded for all samples withdrawn from the bioreactors, and the measurements were performed as reported previously [13]. Details for both spectral measurements are reported in Additional file 1.

### Spectral preprocessing and data analysis

All preprocessing methods and data analyses were performed using Matlab R2022a (The Mathworks Inc., Natick, MA, USA), Unscrambler 11.0 (CAMO Software, Oslo, Norway), and Quasar data mining toolbox version 3.26 (University of Ljubljana, Slovenia) [25, 26].

On-line FT-Raman spectra were baseline-corrected by rubber band baseline correction and smoothed by using the Savitzky-Golay (SG) algorithm (polynomial 2, window size 9, derivative order 0). Afterwards, two datasets referred to as *glass normalisation* and *water normalisation* datasets were created by peak normalisation based on either: (1) a glass-related peak at 800 cm^−1^, or (2) a water-related peak at 3200 cm^−1^, followed by the truncation of data to 3050–2500 and 1800–650 cm^−1^ regions. The third dataset referred to as *EMSC normalisation* dataset (extended multiplicative signal correction (EMSC)) was created by truncation of baseline-corrected and SG-smoothed data to 3050–2500 and 1800–650 cm^−1^ regions, and applying EMSC with linear, quadratic, cubic and quartic terms for *Rhodotorula*, and EMSC with up to sixth order components (linear, quadratic, cubic, quartic, quintic and sextic) for *Schizochytrium*.

At-line HTS-FTIR spectra of both *Rhodotorula* and *Schizochytrium* were converted into second derivatives by using SG algorithm (polynomial order 2, window size 15, derivative order 2), truncation of data to 3050–2800 and 1800–900 cm^−1^ regions, and normalization by EMSC with linear and quadratic components. At-line HTS-FT-Raman spectral data of both *Rhodotorula* and *Schizochytrium* were smoothed by SG algorithm (polynomial 2, window size 9, derivative order 0), followed by the truncation of data to 3050–2500 and 1800–650 cm^−1^ regions, rubber band baseline correction, and correction by EMSC with linear and quadratic components.

Biochemical similarities between samples were estimated by using principal component analysis (PCA) of preprocessed HTS-FTIR and HTS-FT-Raman spectral data.

Pearson correlation coefficients (PCC) test was conducted on the reference data of the online FT-Raman spectral data using both bioreactor replicates, to estimate correlations of measured parameters. This allowed direct comparison of the reference data and analysis of the reproducibility of the fermentation processes.

Partial least squares regression (PLSR) was used to establish calibration models for CDW lipids, carotenoids and carbon substrate. For the on-line FT-Raman spectral data, PLSR models were built by using data of the bioreactor with the FT-Raman spectroscopy flow measurement cell in a recirculatory sampling loop. For the at-line HTS-FTIR and HTS-FT-Raman spectral data, PLSR models were built separately for each bioreactor: one PLSR model for the control and one for the bioreactor with the FT-Raman spectroscopy flow measurement cell in a recirculatory loop. The optimal number of PLSR components (i.e., PLSR factors) of the calibration models (*AOpt*), root-mean-square error (RMSE) and coefficient of determination (R^2^) were calculated in a cross-validation (CV) manner. One-sample-out CV was applied, and the optimal model was selected by optimizing RMSE. PLSR analyses were conducted on all the preprocessed data. The validation of the models built using at-line HTS-FTIR and HTS-FT-Raman spectral data was done on the test sets, that represent independent bioreplicate data, depending on the model: for the model built on the control bioreactor, the validation was done with the data from the bioreactor with the FT-Raman spectroscopy flow measurement cell in a recirculatory loop, and vice versa (R^2^_test_ and RMSE_test_). For the on-line FT-Raman spectral data, only cross-validation performances (R^2^_CV_ and RMSE_CV_) were reported.

### Reference chemical analyses

*Total lipid content and fatty acid profile* were determined by direct transesterification of lipids and their analysis with gas chromatography (GC). Direct transesterification was performed as reported previously [20]. *Carotenoid content and profiles* were determined by high-performance liquid chromatography (HPLC) analysis. The method for the isolation and analysis of carotenoid pigments and ergosterol was adapted from [27]. Details for both chemical analyses are reported in Additional file 1.

*Residual glucose* in the *Rhodotorula* supernatant was analysed by D-Glucose Assay Kit (Megazyme/Neogen, Lansing, MI, USA).

*Residual glycerol* in the *Schizochytrium* supernatant was determined by HLPC analysis using a Dionex Ultimate 3000 system (Dionex) with a Rezex ROA-organic H + (8%) 300 × 7.8 mm analytical column (Phenomenex) and connected to a Shodex RI-101 differential refractive index detector (Shodex). The standards used to interpret the results ranged between 0.5 and 10 g/L glycerol. All samples were diluted 10 times with deionized water (diH_2_O) in a 96-well microtiter filter plate (0.45 μm) using 20 μL sample and 180 μL diH_2_O in each well. The samples were then vacuum filtered and the permeates were used for HPLC analyses. The column temperature was set to 65 °C and 5 mM H_2_SO_4_ was used as eluent with a flow rate of 0.600 mL/min. The HPLC data were recorded and analyzed with the software Chromeleon 7.0 (Thermo Fisher Scientific, Waltham, MA, USA).

## Results and discussion

The online FT-Raman spectroscopy measurements were conducted via non-contact spectral acquisition through the steam sterilized glass flow measurement cell in a recirculatory loop. Since the whole system (comprising the bioreactor and the loop) was autoclaved, the sterile barrier of the bioreactor was preserved during the entire cultivation. Consequently, the measurements were conducted in a non-invasive setting, with no direct contact with the cultivation medium and with minimal risk of contamination. To assess if the recirculatory loop system has significant effect on the fermentation conditions, bioreactor fermentations were carried out in duplicate (with and without recirculatory loop) for both *Rhodotorula* and *Schizochytrium*. In order to minimise pressure and shear stress on the microbial culture and minimize cell damage, we used a Masterflex L/S peristaltic pump with convex roller design Cytoflow 3-roller pump head, which was developed specifically for pumping delicate live cells.

### Fermentations characteristics and chemical analysis of microbial lipids and pigments

**Fig. 1:**
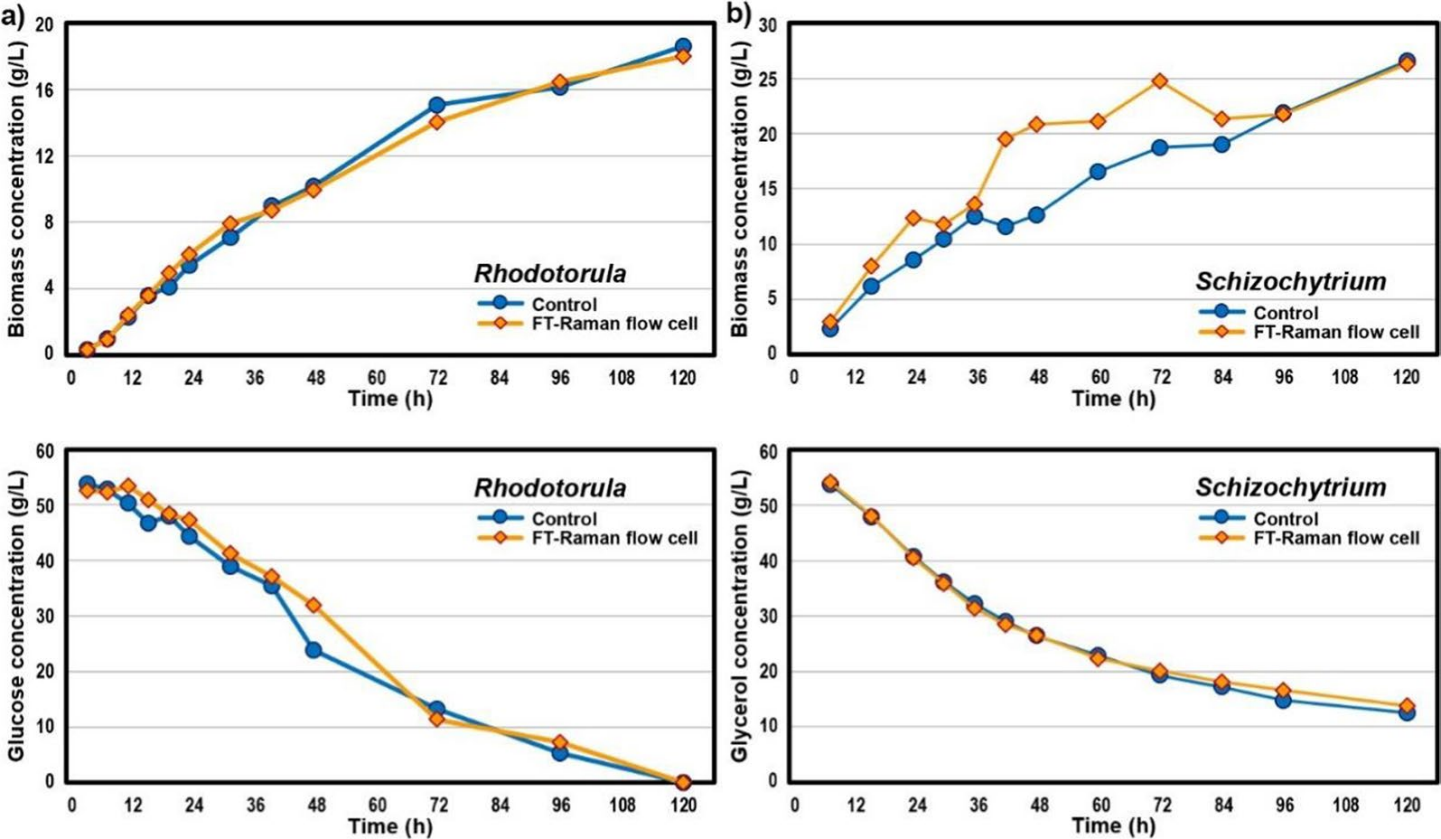
Biomass (**a**, **b**) and carbon substrate (**c**, **d**) concentrations during: (**a**, **c**) fermentation of glucose by *Rhodotorula*, and (**b**, **d**) fermentation of glycerol by *Schizochytrium* for the control bioreactor (blue) and the bioreactor with FT-Raman spectroscopy flow measurement cell in recirculatory loop (orange)

Figure 1 shows data for biomass accumulation and consumption of the carbon source. The obtained final biomass concentrations were 18.3 ± 0.3 g/L of cell dry weight (CDW) for the *Rhodotorula* fermentations, and 26.4 ± 0.2 g/L of CDW for the *Schizochytrium* fermentations. A comparison of carbon substrate concentrations during fermentation for the bioreactor with the FT-Raman spectroscopy flow cell and the control bioreactor shows very high similarity. Glucose was completely consumed by *Rhodotorula* after 120 h, while for *Schizochytrium* fermentations, 77% of the starting glycerol was consumed after 120 h.

**Fig. 2:**
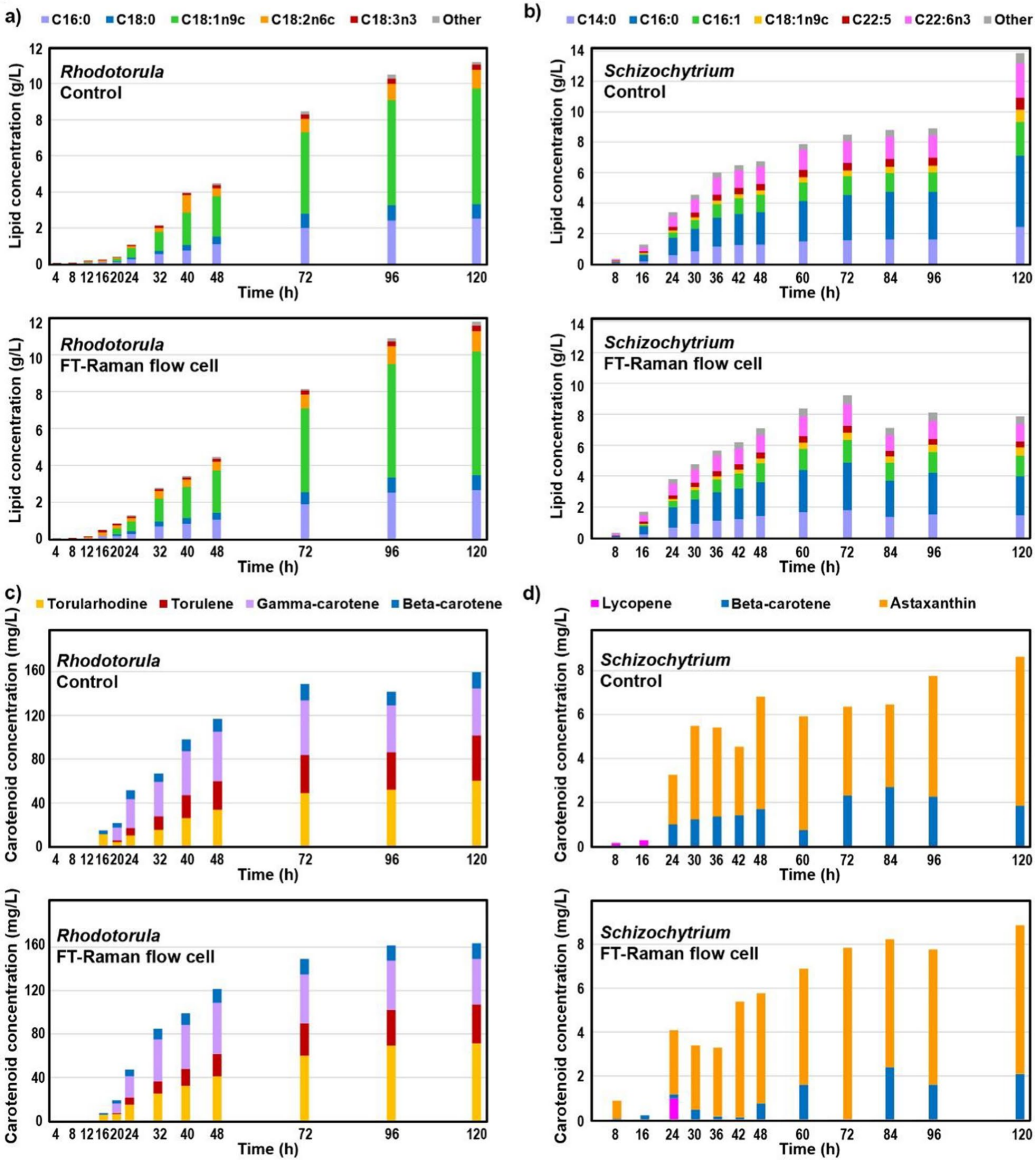
Lipid concentration and fatty acid composition (**a**, **b**), and carotenoid concentration and composition (**c**, **d**) for the control bioreactor and the bioreactor with FT-Raman spectroscopy flow measurement cell in recirculatory loop during: **a**, **c** fermentation of glucose by *Rhodotorula*, and **b**, **d** fermentation of glycerol by *Schizochytrium*

The composition of the microbial biomass was analysed at a range of sampling points. The TAG and FFA lipid concentrations, fatty acid profiles, carotenoid concentrations and profiles are reported in Fig. 2 (relative content in Additional file 1: Fig. S1). *Rhodotorula* reached maximum of TAG and FFA lipid content of 66.3 ± 0.7%_w/w_ after 96 h, and the final content of 66.0 ± 0.5%_w/w_. Fatty acid composition of the lipids was mainly palmitic (C16:0), stearic (C18:0), oleic (C18:1), linoleic (C18:2) and α-linolenic (C18:3) acids. Maximum carotenoid content for *Rhodotorula* fermentation was 12.25 ± 0.35 ‰ _w/w_ (12,252 µg/g) obtained after 48 h, while a final content of 8.98 ± 0.26 ‰ _w/w_ (8985 µg/g) was estimated. Carotenoid composition was mainly *β*-carotene, *γ*-carotene, torulene, and torularhodin. *Schizochytrium* reached a maximum TAG lipid content of 43.7 ± 9.7%_w/w_ after 48 h, and a final content of 41.2 ± 11.1%_w/w_. Fatty acid composition of the lipids was mainly myristic (C14:0), palmitic (C16:0), palmitoleic (C16:1), oleic (C18:1), docosapentaenoic (C22:5) and docosahexaenoic (C22:6) acids. *Schizochytrium* produced relatively small amount of carotenoids (predominantly astaxanthin), with maximum content of 0.40 ± 0.12 ‰ _w/w_ (405 µg/g) obtained after 30 h, and a final content of 0.33 ± 0.01 ‰ _w/w_ (331 µg/g). The results for the biomass, lipid and carotenoid production and composition are in agreement with previously published studies for *R. toruloides* and *Schizochytrium* fermentations [2, 3, 28–30]. Although the fermentation batch processes were not optimized, the accumulation of lipids and carotenoids were relatively high. For example, the obtained carotenoid content for *Rhodotorula* fermentation is one of the highest carotenoids contents for carotenogenic yeasts [3, 30].

While the reproducibility of measurements for biomass and lipid concentrations between the bioreactor with the FT-Raman spectroscopy flow cell and the control bioreactor was high in case of *R. toruloides* fermentations, the reproducibility was lower for *Schizochytrium* fermentations (Figs. 1 and 2). However, excellent reproducibility of glycerol concentrations profiles strongly indicates that the reason for the differences in biomass and lipid concentrations during *Schizochytrium* fermentations may be related to variations in the analysis after the fermentation, and not to differences in the bioprocesses of the two bioreactors (Fig. 1). It is likely that these differences are related to the biomass processing after sampling, possibly related to the difficulty in separating biomass from the cultivation medium due to formation of different layers. It has been noticed previously that separation of thraustochytrid biomass can result in two layers; one layer floating on top of the supernatant composed of zoospores, and a pellet on the bottom composed of oleaginous vegetative cells [31]. The challenge in separation of biomass was causing the general higher standard deviations for *Schizochytrium* fermentations, and resulted in the relatively large deviations observed in the biomass and lipid concentrations. This problem clearly demonstrates the huge value and a need for online noninvasive methods for chemical analysis, and their clear advantage compared to at-line and offline approaches that depend on sampling and multi-step chemical analyses.

Pearson correlation coefficients were used to assess corelations between various fermentation data: (1) concentration data (glucose, glycerol, CDW, total lipids, individual fatty acids (FAs), total carotenoids, and individual carotenoids; all in g/L except carotenoids in mg/L), and (2) relative content (total lipids in %_w/w_, total carotenoids in ‰_w/w_, individual FAs in % of total FAMEs, and individual carotenoids in % of total carotenoids). The correlation matrices showed that all the production parameters are positively correlated, and negatively correlated with the carbon substrates (Additional file 1: Figs. S3–S6). In particular, concentrations of the individual carotenoids are highly correlated with the concentrations of total carotenoids, while concentrations of the individual fatty acids are highly correlated with the concentrations of total TAG and FFA lipids. For this reason we have not reported the regression models for individual carotenoids and fatty acids.

### FT-Raman spectroscopy online monitoring of fermentations

**Fig. 3:**
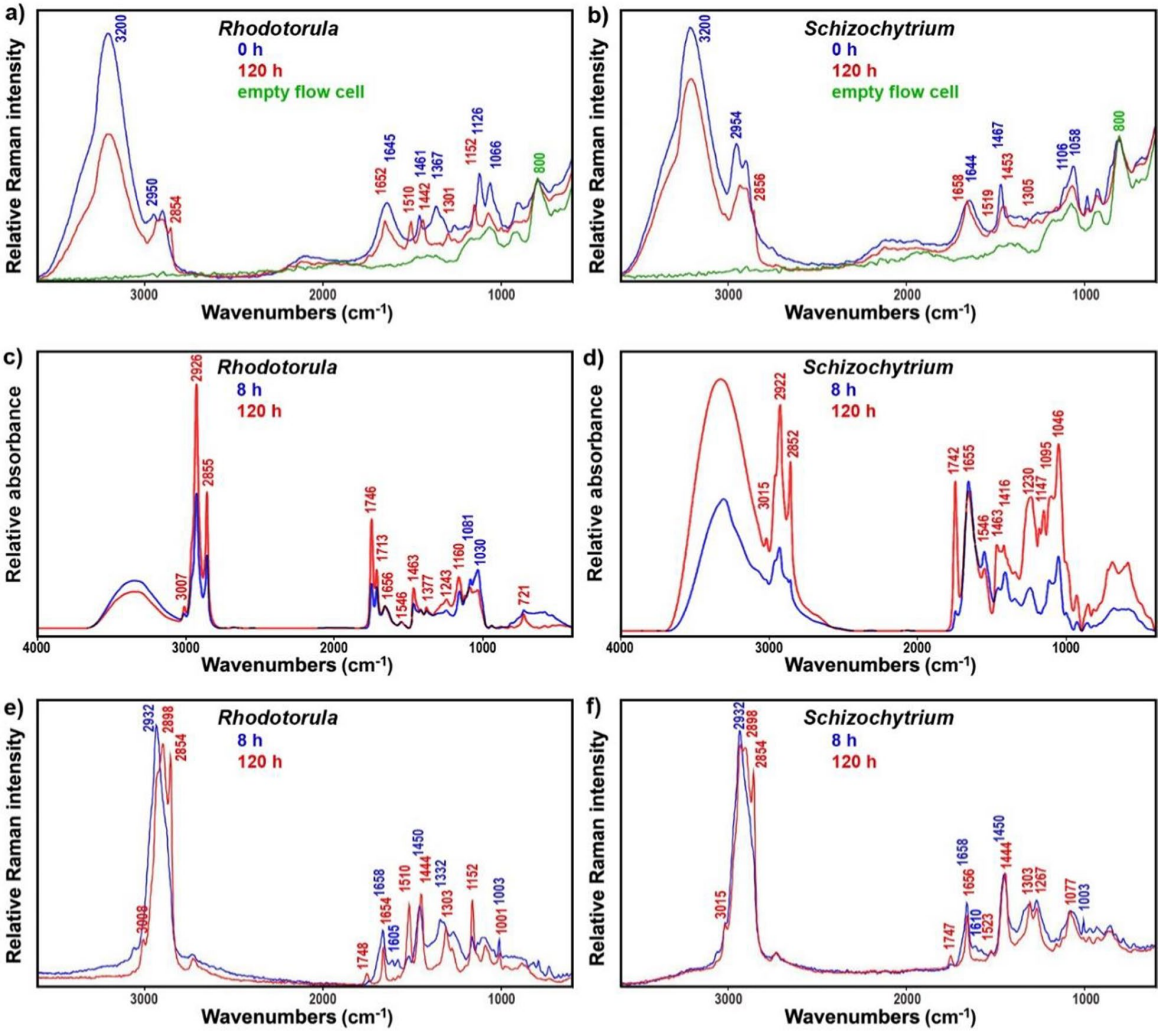
On-line FT-Raman spectra recorded at the start (0 h) and end (120 h) of bioprocesses: **a** fermentation of glucose by *Rhodotorula*, and **b** fermentation of glycerol by *Schizochytrium* (the presented spectra are averages of three consecutive spectra, and were normalised to glass-related peak at 800 cm^−1^; the spectrum of the empty glass flow cell is added for reference). HTS-FTIR (c and d) and HTS-FT-Raman spectra (**e** and **f**) of freeze-dried microbial biomass after 8 h and 120 h of bioprocess: (**c** and **e**) fermentation of glucose by *Rhodotorula*, and (**d** and **f**) fermentation of glycerol by *Schizochytrium* (spectra were baseline corrected and normalised for better viewing)

The representative on-line FT-Raman spectra at the beginning and end of the two fermentations show all characteristic signals of growth media and microbial biomass (Fig. 3a and 3b). The strongest signals at the start of the fermentations are related to carbon substrates: C–H stretching vibrations (2950 and 2896 cm^−1^ C–H stretching of CH_2_ group, for both glucose and glycerol), CH_2_ bending (1467 cm^−1^ for glycerol and 1461 cm^−1^ for glucose), CH_2_ wagging (1370–1320 cm^−1^ for glucose), C–OH stretching and CH_2_ twisting (1120–1050 cm^−1^ for glycerol), C–OH stretching and bending (1130–1020 cm^−1^ for glucose), and skeletal C–C stretch (860 cm^−1^ for glycerol) [32, 33]. In addition, the spectra are dominated by the signals related to O–H stretching and H–O–H bending of water (at 3400–3000 cm^−1^ and 1645 cm^−1^, respectively), as well as Si–O and O–Si–O stretching of borosilicate glass flow cell (at 1100–900 and 800 cm^−1^, respectively) [34]. The end spectra show strong signals related to lipids (in particular C–H stretching in –CH_2_ groups at 2855 cm^−1^, C=C stretching at 1655 cm^−1^, CH_2_/CH_3_ scissoring around 1448 cm^−1^, and CH_2_ twisting at 1303 cm^−1^) and carotenoids (in particular C=C stretching around 1515 cm^−1^, and C–C stretching and C–H deformation at 1152 cm^−1^).

The online FT-Raman spectroscopy setup was assessed and compared to the at-line high throughput FTIR and FT-Raman spectroscopies (HTS). The HTS spectra contain rich information on intracellular metabolites (Fig. 3c–f). In general, the most intensive bands are associated with TAG and FFA lipids (approx. peak positions in parentheses): C–H stretching vibrations (=C–H stretching at 3010 cm^−1^; C–H stretching in –CH_3_ and –CH_2_ at 2925, 2898 and 2855 cm^−1^), C=O stretching in esters (1746 cm^−1^), C=O stretching in fatty acids (1713 cm^−1^, only in FTIR), C=C stretching (1655 cm^−1^, only in FT-Raman spectra), CH_2_ and CH_3_ deformations (1463 cm^−1^ in FTIR, and 1444, and 1303 cm^−1^ in FT-Raman spectra) [35]. In addition, the HTS spectra show intensive bands associated with proteins: C=O stretching in amides (1658 cm^−1^, Amide I), CNH deformations (1546 cm^−1^, Amide II, only in FTIR spectra), phenyl ring C=C stretching (1608 cm^−1^, only in FT-Raman spectra) and deformations in tyrosine and phenylalanine (1003 cm^−1^, only in FT-Raman spectra) [13]. Furthermore, the HTS-FTIR spectra show bands associated with phosphates, namely P=O stretching (approx. 1235 cm^−1^), as well as β-glucans in the spectra of *Rhodotorula*, namely CH_2_ and CH_3_ deformations (1332 cm^−1^), C–C, C–O, C–O–C, CH, COH stretching, deformations and combination bands (1081 and 1030 cm^−1^) [13]. Finally, FT-Raman spectra show bands associated with carotenoids that are detectable due to strong resonance Raman effect of carotenoids caused by strong electron–phonon coupling: –C=C– stretching (at approx. 1515 cm^−1^), –C–C– stretching and CH deformation (1152 cm^−1^), and C–CH_3_ deformations (1005 cm^−1^) [36].

The PLSR models were obtained for quantitative estimates of carbon substrate (glucose and glycerol, both in g/L), CDW (g/L), total TAG and FFA lipids (g/L), and total carotenoids (mg/L) concentrations, as well as for relative contents of total lipids (%_w/w_), and total carotenoids (‰_w/w_). For building the regression models we used different normalisation approaches. Specifically, water- and glass-specific signals were used as external and internal standards in the normalisation step of preprocessing, in addition to standard EMSC preprocessing, to create different spectral datasets (Table 1). These specific signals of the glass flow cell and water can be of high importance to obtain accurate concentration values of the production parameters (biomass, carbon substrates, lipids and pigments), since they enable compensation for the variations in Raman spectral intensities due to changing experimental conditions. For example, changes in cell growth (i.e. increased turbidity of growth media) and cell morphology during a life cycle cause variations in light scattering. Other variations could be caused by time-dependent changes in fluorescence interference, and instrumental imperfections, such as variation in laser output power.

**Table 1:** PLSR analysis of on-line data

Analysis	Range	EMSC		Water normalisation		Glass normalisation	
		R^2^_CV_ (*A*)	RMSE_CV_	R^2^_CV_ (*A*)	RMSE_CV_	R^2^_CV_ (*A*)	RMSE_CV_
*Rhodotorula*							
Glucose (g/L)	0.03–52.54	0.99 (2)	2.16 (4.1%)	0.99 (2)	1.78 (3.4%)	0.97 (2)	3.12 (5.9%)
Cell dry weight (g/L)	0.32–18.01	0.99 (1)	0.62 (3.4%)	0.98 (1)	0.84 (4.7%)	0.99 (2)	0.65 (3.6%)
Total lipids (%_w/w_)	5.57–67.04	0.97 (1)	3.81 (5.7%)	0.95 (1)	4.98 (7.4%)	0.97 (2)	3.84 (5.9%)
Total lipids (g/L)	0.02–11.98	0.99 (2)	0.41 (3.4%)	0.99 (2)	0.38 (3.2%)	0.97 (2)	0.78 (6.5%)
Total carotenoids (‰_w/w_)	0–12.62	0.88 (2)	1.66 (13.1%)	0.85 (2)	1.83 (14.5%)	0.71 (1)	2.59 (20.5%)
Total carotenoids (mg/L)	0–166.51	0.94 (1)	16.09 (9.7%)	0.91 (1)	19.42 (11.7%)	0.94 (1)	15.70 (9.4%)
*Schizochytrium*							
Glycerol (g/L)	13.86–54.17	0.99 (1)	1.13 (2.1%)	0.99 (1)	1.49 (2.8%)	0.99 (2)	1.15 (2.1%)
Cell dry weight (g/L)	2.98–26.31	0.91 (1)	2.05 (7.7%)	0.91 (1)	2.05 (7.7%)	0.87 (1)	2.46 (9.6%)
Total lipids (%_w/w_)	10.87–41.50	0.20 (1)	7.71 (18.5%)	0.26 (1)	7.33 (17.7%)	0.41 (1)	6.48 (15.6%)
Total lipids (g/L)	0.32–9.22	0.92 (1)	0.76 (8.2%)	0.94 (1)	0.67 (7.2%)	0.93 (1)	0.68 (7.4%)
Total carotenoids (‰_w/w_)	0.03–0.39	0.03 (1)	0.10 (25.6%)	0.06 (1)	0.07 (17.9%)	0.01 (1)	0.11 (28.2%)
Total carotenoids (mg/L)	0.21–8.87	0.89 (1)	0.93 (10.5%)	0.85 (1)	1.07 (12.1%)	0.79 (1)	1.30 (14.7%)

Coefficients of determination (R^2^) and root mean square errors (RMSE) of prediction for determination of glucose/glycerol, cell dry weight, total FAME lipids, and total carotenoids, with the number of components in parenthesis (*Aopt*), for the regression analyses based on three different types of spectral preprocessing. The R^2^ and RMSE of cross validation are calculated for the models built on the data of the bioreactor with FT-Raman spectroscopy flow measurement cell in recirculatory loop

**Fig. 4:**
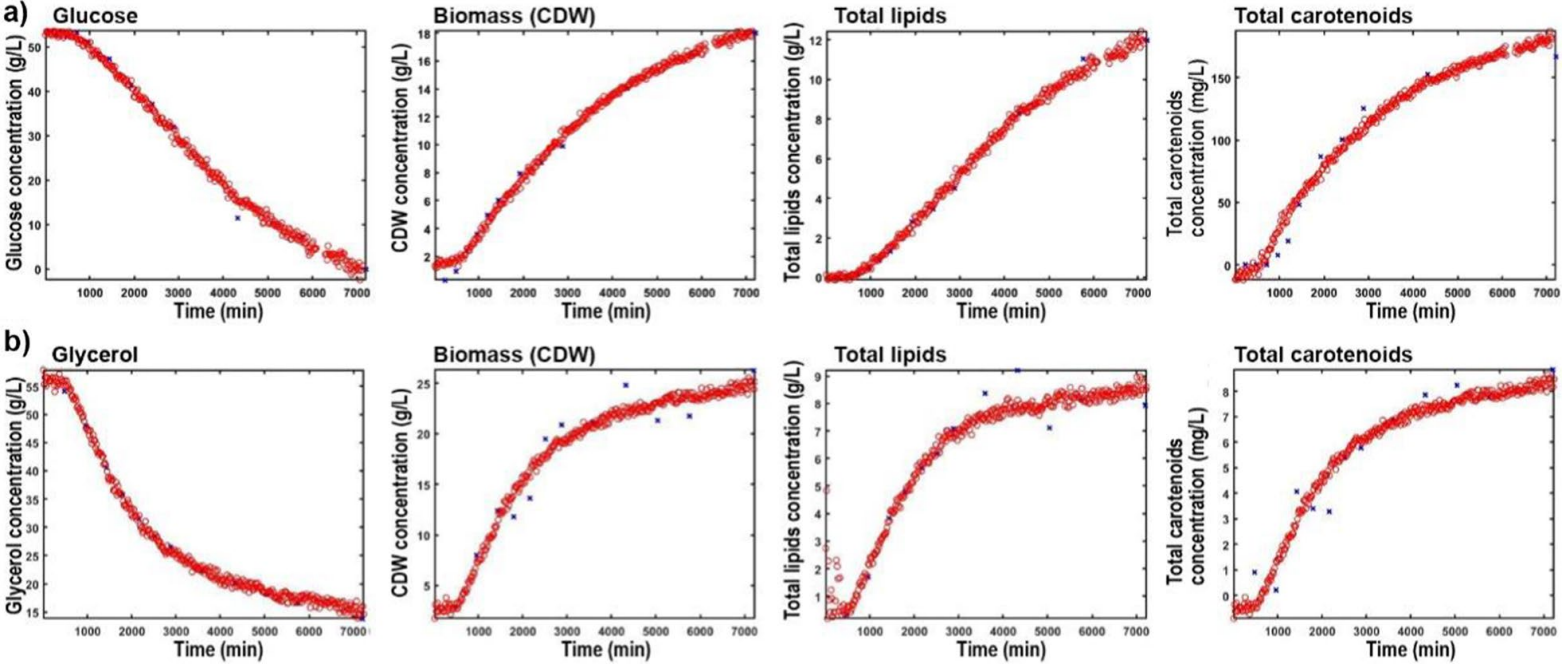
Concentration profiles for carbon substrate, biomass (CDW), total FAME lipids and total carotenoids (red ○), based on the best PLSR models established on regression of spectral data in selected timepoints on off-line reference data (blue ×), for: **a** fermentation of glucose by *Rhodotorula* and **b** fermentation of glycerol by *Schizochytrium*

The PLSR models (Table 1, Fig. 4 and Additional file 1: Figs. S7 and S8) show high level of correlation between the online FT-Raman spectroscopy data and reference measurements, with coefficients of determination (R^2^) in 0.94–0.99 range for all concentration parameters of *Rhodotorula* fermentation, and 0.89–0.99 range for the corresponding parameters of *Schizochytrium* fermentation (Table 1). The models for all concentration parameters were built with up to two PLS components, signifying stability and reliability of the models. The PLS factors for *Rhodotorula* fermentation show strong contributions of signals related to glucose (1363 and 1127 cm^−1^), lipids (2927, 2853, 1745, 1655, and 1440 cm^−1^), carotenoids (1510 and 1153 cm^−1^), and glass (800 cm^−1^). The PLS factors for *Schizochytrium* fermentation show strong contributions of signals related to glycerol (2954, 1469, 1056 and 860 cm^−1^), lipids (2920, 2858, 1658, and 1442, 1303 cm^−1^), and carotenoids (1520 and 1153 cm^−1^).

The RMSE relative values for all concentration parameters of the *Rhodotorula* fermentation were within 3.2–9.4% range (for the best PLSR models), with very similar values to previously published studies employing online Raman spectroscopy monitoring of yeast (*Saccharomyces cerevisiae* and *Cutaneotrichosporon oleaginosus*) fermentations. For example, the RMSE for biomass concentration was 0.62 g/L, well within the published range of 0.30–0.96 g/L, while the RMSE for glucose concentration was 1.78 g/L, again well within the published range of 0.92–4.78 [6–8, 10, 14]. As RMSE value for carotenoids concentration we obtained 15.70 mg/L (9.4%) while the concentration range was relatively broad (0–166.51 mg/L). This is a significantly higher error than the RMSE obtained for monitoring of carotenoids concentration for the carotenogenic yeast *Phaffia rhodozyma* by a online Raman spectroscopy system (RSME = 2.3 mg/L), which is to our knowledge the only other report on online Raman spectroscopy monitoring of carotenoids [37]. However, the concentration range in the study of Cannizzaro et al. was considerably smaller and in the range of 0–45 mg/L. Moreover, due to the extremely strong resonant Raman signals of carotenoids that mask completely the regular Raman spectral contributions from other compounds, none of the other critical concentration parameters (such as carbon substrate or biomass) were obtainable for evaluation in the study of Cannizzaro et al. Thus, this clearly shows the value of our approach with an FT-Raman spectroscopy system employing a NIR laser with a 1064 nm excitation wavelength, that provides spectra with relatively weak resonant Raman spectral signals and strong signals of other analytes of interest. Regarding lipid concentrations, to our knowledge, our study is the first example of monitoring concentration of lipids by an online Raman spectroscopy system, and we obtained a very low RMSE of only 0.38 g/L (3.2%). To our knowledge the only comparable study by [6] obtained an RMSE of 3.53%_w/w_ for measurement of relative lipid content, which is very similar to our RMSE value for the same parameter (3.81%_w/w_; 5.7%). For the *Schizochytrium* fermentation, the RMSE values for all concentration parameters were within a 2.1–10.5% range (for the best PLSR models). This is in general slightly higher than the RMSEs obtained for the corresponding parameters during *Rhodotorula* fermentation. To our knowledge, this is the first study where thraustochytrid fermentation was monitored by a vibrational spectroscopy PAT system, including all types of online, at-line and off-line FTIR and Raman spectroscopy systems. Since the RMSE for glycerol concentration was extremely low with a value of just 1.13 g/L (2.1%), it can be assumed that the main contribution to the error of the PLS models for other concentration parameters comes from the measurement error in the reference data, and is not due to variations in the online FT-Raman spectral data. It is important to emphasize that all reference methods introduce error through sampling procedures, they require large amount of biomass which prevents measurement of technical replicates, and they also include several processing steps (including wet chemistry) that almost certainly increase errors even further. Finally, the PLSR results in Table 1 clearly show that regression models for different types of analytes often require different spectral preprocessing methods, as we already elaborated in the earlier study [38].

### Off-line (at-line) FTIR and FT-Raman spectroscopy monitoring of fermentations

Although the HTS-spectroscopy measurements in this study were conducted in off-line mode on freeze-dried biomass, the same approach can be conducted in at-line mode on wet biomass. HTS-FTIR and HTS-FT-Raman spectra of wet biomass can be obtained in approx. 1–2 h after sampling, depending on sample preparation and measurement parameters. Therefore, this approach allows cost-efficient at-line monitoring of fermentation, albeit with modest time delay and other aforementioned drawbacks of sampling at defined timepoints. The representative HTS-FTIR and HTS-FT-Raman spectra of freeze-dried microbial biomass at the beginning (after 8 h) and end (120 h) of bioprocesses are depicted in Fig. 3.

The scores plots of the principal component analysis (PCA) of HTS-FTIR and HTS-FT-Raman spectral data show that the differences in chemical composition of microbial biomass between the bioprocesses conducted in the control bioreactor and the bioreactor with a recirculatory loop are relatively small (Additional file 1: Fig. S9). The loading plots show that the spectra of the starting biomasses are dominated by signals related to proteins, followed by stronger signals related to cell wall carbohydrates in the first 24 h of the fermentations, and finally gradual increase during 24–120 h of signals related to TAG lipids, FFA lipids (only for *Rhodotorula*), and carotenoids (Additional file 1: Fig. S10).

We have shown previously that PLSR of HTS-FTIR and HTS-FT-Raman spectral data can provide accurate assessments of intra- and extracellular microbial metabolites [13, 18, 19, 21, 23, 24, 39]. The RMSE values for cross validation were within a range of 4–20% for fermentation of glucose by *Rhodotorula*, and 4–44% for fermentation of glycerol by *Schizochytrium* (Tables 2 and 3, respectively, and Additional file 1: Figs. S11–S14). The RMSE values for independent validation were within a range of 5–20% for *Rhodotorula*, and 11–26% for *Schizochytrium* (Additional file 1: Table S1). It is important to note that the spectral datasets only enable direct measurement of relative contents of total lipids (for both HTS-FTIR and HTS-FT-Raman spectroscopy), and total carotenoids (only for HTS-FT-Raman spectroscopy) since concentration parameters can be obtained only indirectly from measurements of biomass isolated from a fermentation broth. Unsurprisingly, the PLSR models based on offline spectral data for quantitative estimates of carbon substrate, CDW, total lipids, and total carotenoids concentrations had higher complexity and worse performance than their counterparts based on online FT-Raman spectral data. In general, the PLSR models based on the online data are very robust, comprising only 1–2 PLSR components, compared to higher complexity of models based on the offline spectral data (2–4 components). Moreover, since these offline data prediction models depend on indirect measurements of concentration parameters, it can be expected that their performance worsens dramatically for fed-batch and continuous fermentations, were biomass and carbon substrate concentrations will not be so highly correlated. The PLSR models based on offline spectral data for quantitative estimates of relative contents of total lipids (%_w/w_), and total carotenoids (‰_w/w_) had better performance, similar to the corresponding models based on online FT-Raman spectral data. The RMSE values for cross validation for assessment of relative contents of total lipids in *Rhodotorula* biomass were approx. 5 and 8% for the models based on HTS-FT-Raman and HTS-FTIR spectral data, respectively, while for *Schizochytrium* biomass the corresponding errors were slightly higher (approx. 9 and 11%, respectively). For the models based on HTS-FT-Raman spectral data, the RMSE values for assessment of relative contents of carotenoids in *Rhodotorula* and *Schizochytrium* biomass were approx. 13 and 27%, respectively.

**Table 2:** PLSR analysis of offline (at-line) HTS-FT-Raman spectral data

Analysis	Control			Bypass loop		
	Range	R^2^_CV_ (*A*)	RMSE	Range	R^2^_CV_ (*A*)	RMSE
*Rhodotorula*						
Glucose (g/L)	0.04–53.81	0.93 (2)	4.86 (9.0%)	0.03–52.54	0.91 (2)	5.65 (10.7%)
Cell dry weight (g/L)	0.32–18.60	0.94 (2)	1.47 (7.9%)	0.32–18.01	0.93 (2)	1.47 (8.2%)
Total lipids (%_w/w_)	4.96–65.54	0.98 (3)	3.08 (4.7%)	5.57–67.04	0.98 (3)	3.24 (4.8%)
Total lipids (g/L)	0.02–12.18	0.90 (2)	1.33 (10.9%)	0.02–11.98	0.88 (2)	1.42 (11.9%)
Total carotenoids (‰_w/w_)	0–11.88	0.88 (2)	1.51 (12.7%)	0–12.62	0.88 (1)	1.66 (13.1%)
Total carotenoids (mg/L)	0–162.23	0.97 (2)	11.06 (6.8%)	0–166.51	0.99 (3)	6.3 (3.8%)
*Schizochytrium*						
Glycerol (g/L)	12.53–53.75	0.93 (4)	3.29 (6.1%)	13.86–54.17	0.94 (4)	2.78 (5.1%)
Cell dry weight (g/L)	2.36–26.56	0.76 (2)	3.23 (12.2%)	2.98–26.31	0.90 (4)	2.05 (7.8%)
Total lipids (%_w/w_)	13.97–56.13	0.89 (4)	4.48 (8.0%)	10.87–41.50	0.60 (1)	3.99 (9.6%)
Total lipids (g/L)	0.33–13.87	0.76 (2)	1.81 (13.1%)	0.32–9.22	0.87 (4)	1.44 (15.6%)
Total carotenoids (‰_w/w_)	0.05–0.54	0.50 (1)	0.11 (20.4%)	0.03–0.39	0.19 (1)	0.15 (38.5%)
Total carotenoids (mg/L)	0.16–8.63	0.81 (3)	1.15 (17.5%)	0.21–8.87	0.81 (5)	0.97 (10.9%)

Coefficients of determination (R^2^) and root mean square errors (RMSE) of prediction for determination of glucose/glycerol, cell dry weight, total FAME lipids, and total carotenoids, with the number of components in parenthesis (*Aopt*), for the regression analyses with cross validation. The PLSR models were built on the data of the control bioreactor and of the bioreactor with on-line FT-Raman flow measurement cell in recirculatory loop (bypass loop)

**Table 3:** PLSR analysis of offline (at-line) HTS-FTIR spectral data

Analysis	Control			Bypass loop		
	Range	R^2^_CV_ (*A*)	RMSE	Range	R^2^_CV_ (*A*)	RMSE
*Rhodotorula*						
Glucose (g/L)	0.04–53.81	0.83 (2)	7.58 (14.1%)	0.03–52.54	0.72 (2)	9.88 (18.6%)
Cell dry weight (g/L)	0.32–18.60	0.87 (2)	2.15 (11.6%)	0.32–18.01	0.81 (2)	2.39 (13.3%)
Total lipids (%_w/w_)	4.96–65.54	0.97 (2)	4.38 (6.7%)	5.57–67.04	0.92 (2)	6.3 (9.4%)
Total lipids (g/L)	0.02–12.18	0.80 (2)	1.94 (15.9%)	0.02–11.98	0.69 (2)	2.33 (19.4%)
Total carotenoids (‰^w/w^)	0–11.88	0.80 (1)	1.96 (16.5%)	0–12.62	0.76 (2)	2.35 (18.6%)
Total carotenoids (mg/L)	0–162.23	0.96 (2)	12.40 (7.6%)	0–166.51	0.96 (2)	12.40 (7.4%)
*Schizochytrium*						
Glycerol (g/L)	12.53–53.75	0.94 (3)	3.13 (5.8%)	13.86–54.17	0.98 (5)	2.00 (3.7%)
Cell dry weight (g/L)	2.36–26.56	0.93 (5)	1.78 (6.7%)	2.98–26.31	0.95 (3)	1.51 (5.7%)
Total lipids (%^w/w^)	13.97–56.13	0.73(1)	6.57 (11.7%)	10.87–41.50	0.73 (1)	4.64 (11.2%)
Total lipids (g/L)	0.33–13.87	0.93 (8)	1.01 (7.2%)	0.32–9.22	0.79 (2)	1.24 (13.4%)
Total carotenoids (‰_w/w_)	0.05–0.54	0.42 (1)	0.12 (22.2%)	0.03–0.39	0.05 (1)	0.17 (43.6%)
Total carotenoids (mg/L)	0.16–8.63	0.83 (3)	1.36 (15.7%)	0.21–8.87	0.85 (4)	1.24 (14.0%)

Coefficients of determination (R^2^) and root mean square errors (RMSE) of prediction for determination of glucose/glycerol, cell dry weight, total FAME lipids, and total carotenoids, with the number of components in parenthesis (*Aopt*), for the regression analyses with cross validation. The PLSR models were built on the data of the control bioreactor and of the bioreactor with on-line FT-Raman spectroscopy flow measurement cell in recirculatory loop (bypass loop)

## Conclusions

For fermentations of oleaginous and carotenogenic microorganisms, it is vital to monitor the substrate consumption and changes in lipid and pigment content in order to optimize the process and determine optimal harvesting time for each type of product. The FT-Raman spectrometer with a flow cell in a recirculatory loop can precisely measure in real-time characteristic signals related to carbon substrates, TAG and FFA lipids and carotenoid pigments, irrespective of carbon substrate or microbial type. In general, this approach is superior to the at-line HTS-FT-Raman and HTS-FTIR spectroscopy methods since the obtained information is more comprehensive, timely and provides more precise concentration profiles. The dynamic production profiles (Additional file 1: Fig. S15) can enhance process understanding, and provide vital input parameters for process modeling and optimisation. In addition, such an approach can be utilized in fed-batch and continuous fermentations. Ultimately, this PAT approach will allow timely monitoring of critical process parameters and continuous quality control of process products.

The FT-Raman spectroscopy system with a flow measurement cell in a recirculatory loop, in combination with PLSR prediction models, can simultaneously provide real-time concentration data on carbon substrate utilization, and production of biomass, carotenoid pigments, and lipids (TAG and FFA). This data enables monitoring of dynamic behavior of oleaginous and carotenogenic microorganisms, and thus can provide critical process parameters for process optimization and control. The study has presented broad and global appeal of this noninvasive FT-Raman spectroscopy-based PAT system by obtaining concentration parameters from fermentations utilizing different type of microorganisms (yeast and thraustochytrid) and different type of carbon substrates (glucose and glycerol).

### Supplementary Information

**Additional file 1:** The supplementary file includes supplementary details on sample measurements, chemical analyses, and data analyses.

## Data Availability

The datasets used in this study are available from the corresponding author on reasonable request.
