# Supplementary material for: Raman spectroscopy online monitoring of biomass production, intracellular metabolites and carbon substrates during submerged fermentation of oleaginous and carotenogenic microorganisms
Source: Microb Cell Fact. 2023 Dec 18;22:261. doi: 10.1186/s12934-023-02268-y (PMC10729511; doi:10.1186/s12934-023-02268-y)
Supplement: Supplementary file 1 — Additional file 1: The supplementary file includes supplementary details on sample measurements, chemical analyses, and data analyses. [file 12934_2023_2268_MOESM1_ESM.pdf]

Supplementary Material

Raman spectroscopy online monitoring of biomass production, intracellular metabolites and carbon substrates during submerged fermentation of oleaginous and carotenogenic microorganisms

Simona Dzurendová[1] (simona.dzurendova@nmbu.no), Pernille Margrethe Olsen[2] (pernille.margrethe.olsen@nmbu.no), Dana Byrtusová[1] (dana.byrtusova@nmbu.no), Valeria Tafintseva[1] (valeria.tafintseva@nmbu.no), Volha Shapaval[1] (volha.shapaval@nmbu.no), Svein Jarle Horn[2] (svein.horn@nmbu.no), Achim Kohler[1] (achim.kohler@nmbu.no), Martin Szotkowski[3] (szotkowski@fch.vut.cz), Ivana Marova[3] (marova@fch.vut.cz), Boris Zimmermann[1]* (boris.zimmermann@nmbu.no)

[1]Faculty of Science and Technology, Norwegian University of Life Sciences, P.O. Box 5003, N-1432 Ås, Norway
[2]Faculty of Chemistry, Biotechnology and Food Science, Norwegian University of Life Sciences, P.O. Box 5003, N-1432 Ås, Norway
[3]Institute of Food Science and Biotechnology, Faculty of Chemistry, Brno University of Technology, Purkyňova 464/118, 61200 Brno, Czech Republic

*Corresponding author:
Boris Zimmermann
Faculty of Science and Technology
Norwegian University of Life Sciences
Drøbakveien 31, 1432 Ås, Norway.
Tel: +47 6723 1576
Fax: +47 6496 5001
E-mail: boris.zimmermann@nmbu.no

Table of Contents **Page**

Chemical analysis: Total lipid content and fatty acid profile S-2
Chemical analysis: Carotenoid content and profiles S-3
HTS-FT-Raman and HTS-FTIR measurements S-3
Figure S1. TAG and FFA lipid production S-4
Figure S2. Carotenoid production S-4
Figure S3-S6. Correlation matrices of production parameters S-5
Figure S7-S8. PLSR analysis of on-line spectroscopy data S-9
Figure S9-S10. PCA score and loading plots for at-line spectral data S-11
Table S1. PLSR results for at-line HTS spectroscopy S-13
Figure S11-S14. PLSR coefficients for at-line spectral data S-14
Figure S15. Production rates for fermentation of glucose by *Rhodotorula* S-18

Total lipid content and fatty acid profile were determined by direct transesterification of lipids and their analysis with gas chromatography (GC). Direct transesterification was performed according to Lewis et al. (Lewis et al., 2000), with modifications as reported previously (Langseter et al., 2021).

2 mL screw-cap polypropylene (PP) tube was filled with approx. 20 mg freeze-dried biomass, approx. 250 mg (710–1180 µm diameter) acid-washed glass beads (Sigma-Aldrich, USA), 1 mg of TAG internal standard solution in hexane (100 µL from a 10 mg/mL glyceryl tritridecanoate; $C_{42}H_{80}O_6$, TG(13:0/13:0/13:0), Sigma-Aldrich, USA), and 500 µL chloroform. Biomass was processed in a Precellys Evolution tissue homogenizer (Bertin Instruments, France) at 5500 rpm, for 20 s cycle length and 6 cycles (6×20 s). The processed biomass was transferred into glass reaction tube by washing the PP tube with 2400 µL methanol–chloroform–hydrochloric acid solvent mixture (7.6:1:1 v/v) (3×800 µL). Finally, 500 µL of methanol was added into glass reaction tube. The reaction mixture was incubated at 90 °C for 90 min, followed by cooling to room temperature. 1 mL of distilled water was added. The fatty acid methyl esters (FAMEs) were extracted by the addition of 2 mL hexane followed by 10 s vortex mixing. The reaction tubes were centrifuged at 3000 rpm for 5 min at 4 °C, and the upper organic phase was collected in glass tubes. The lower (water phase) was extracted two more times, but now by the addition of 2 mL hexane–chloroform mixture (4:1 v/v). The solvent in glass tubes was evaporated under nitrogen at 30 °C. Small amount of anhydrous sodium sulfate was added in glass tubes. FAMEs were transferred into GC vials by washing the glass tube with 1500 µL hexane (2×750 µL) containing 0.01% butylated hydroxytoluene (BHT, Sigma-Aldrich, USA) followed by 5 s vortex mixing (slow speed). Determination of total lipid content (expressed as the wt% of total fatty acid methyl esters (FAMEs) of sample dry weight) and fatty acid composition (expressed as wt% of individual FAME of total FAMEs) were performed by using gas chromatography 7820A System (Agilent Technologies, USA), equipped with an Agilent J&W 121-2323 DB-23 column, 20m × 180 µm × 0.20 µm and a flame ionization detector (FID). Helium was used as a carrier gas. The total runtime for one sample was 36 minutes with the following oven temperature increase: initial temperature 70 °C for 2 minutes, after 8 minutes to 150 °C with no hold time, 230 °C in 16 minutes with 5 minutes hold time, and 245 °C in one minute with 4 minutes hold time. The injector temperature was 250 °C and 1 µl of a sample was injected (30:1 split ratio, with split flow 30 mL/min). For the identification and quantification of fatty acids, the Supelco 37 Component FAME Mix (C4–C24 FAME mixture, Sigma-Aldrich, USA) was used as an external standard, in addition to C13:0 TAG internal standard. Measurements were controlled by the Agilent OpenLAB software (Agilent Technologies, USA).

Langseter, A.M., Dzurendova, S., Shapaval, V., Kohler, A., Ekeberg, D., Zimmermann, B. 2021. Evaluation and optimisation of direct transesterification methods for the assessment of lipid accumulation in oleaginous filamentous fungi. Microbial Cell Factories, 20(1).

Lewis, T., Nichols, P.D., McMeekin, T.A. 2000. Evaluation of extraction methods for recovery of fatty acids from lipid-producing microheterotrophs. Journal of Microbiological Methods, 43(2), 107-116.

Carotenoid content and profiles were determined by high-performance liquid chromatography (HPLC) analysis. The method for the isolation and analysis of carotenoid pigments and ergosterol was adapted from Szotkowski et al. (Szotkowski et al., 2019).

15 ± 3 mg of freeze-dried biomass was weighed into plastic extraction tubes and rehydrated by the addition of 1 mL of distilled water. The water was removed by centrifugation (10,000 rpm/5 min/10 °C), and 300 ± 20 mg of acid-washed glass beads (250–500 μm diameter, Roth, Germany) and 1 mL of methanol were added to the pellet. The samples were vortexed for 10 min (2500 rpm). The content of the PP tube was transferred into a glass reaction tube by washing with a 2000 μL of chloroform and the glass tube was vortexed for another 10 min. 1 mL of distilled water was added for the phase separation. After centrifugation (3000 rpm/5 min/4 °C), the bottom chloroform phase containing the extracted pigments and ergosterol was separated and evaporated under nitrogen at 25 °C, followed by the addition of 1 mL of a mixture of ethylacetate:acetonitrile (20:60). The ethylacetate:acetonitrile mixture, containing extracted pigments, was filtered through a syringe filter (0.45 µm, PTFE membrane, 13 mm) and transferred into glass vials for HPLC analysis. The mobile phases consist of mixture A (84% of acetonitrile, 2% of methanol and 14% 0.1 M Tris-HCl (pH = 8)) and mixture B (68% of methanol and 32% of ethylacetate). The contents of individual pigments (beta-carotene, lycopene, torulene, torularhodin) were calculated using calibration standards. Thermo Finnigan Surveyor HPLC/PDA system (Thermo Fisher Scientific, Waltham, MA, USA) and Xcalibur software was used for chromatography data analysis. Beta-carotene and lycopene were purchased from Sigma Aldrich/Merck, Germany, while torularhodin and torulene from CaroteNature, Ltd., Switzerland.

Szotkowski, M., Byrtusova, D., Haronikova, A., Vysoka, M., Rapta, M., Shapaval, V., Marova, I. 2019. Study of Metabolic Adaptation of Red Yeasts to Waste Animal Fat Substrate. Microorganisms, 7(11).

At-line HTS-FT-Raman spectra were recorded for all samples withdrawn from the bioreactors in backscattering geometry using MultiRAM FT-Raman spectrometer (Bruker Optik GmbH, Germany). For each measurement, 2-3 mg of freeze-dried sample was deposited in Agilent 400 µL flat bottom glass vial insert, and inserts were placed in the high throughput sample stage. The spectra were recorded with a total of 512 (*Schizochytrium*) or 2048 (*Rhodotorula*) scans, using Norton-Beer medium apodization, spectral resolution of 8 cm^{-1}, with a digital resolution of 1.928 cm^{-1}, over the range of 3785-45 cm^{-1}, at 500 (*Rhodotorula*) or 1000 mW (*Schizochytrium*) laser power. Each biomass sample was analysed in three technical replicates. The OPUS software (Bruker Optik GmbH, Germany) was used for data acquisition and instrument control.

At-line HTS-FTIR transmittance spectra were measured for all samples withdrawn from the bioreactors using the high throughput screening extension unit (HTS-XT) coupled to the Vertex 70 FTIR spectrometer (both Bruker Optik, Germany). The FTIR system was equipped with a globar mid-IR source and a deuterated L-alanine doped triglycine sulfate (DLaTGS) detector. 2-3 mg of washed freeze-dried biomass was resuspended in 0.5 mL of distilled water. 4 µL of washed cells suspension was pipetted onto an IR transparent 384-well silica microplate, and dried at room temperature for two hours. The HTS-FTIR spectra were recorded with a total of 64 scans, using Blackman-Harris 3-Term apodization, spectral resolution of 6 cm^{-1}, and digital spacing of 1.928 cm^{-1}, over the range of 4000–400 cm^{-1}, and an aperture of 6 mm. Spectra were recorded as the ratio of the sample spectrum to the spectrum of the empty IR transparent microplate. Each biomass sample was analysed in three technical replicates, except the sample from the bioreactor with FT-Raman flow measurement cell at 8 h timepoint, which was not acquired due to a measurement error. The OPUS software (Bruker Optik GmbH, Germany) was used for data acquisition and instrument control.

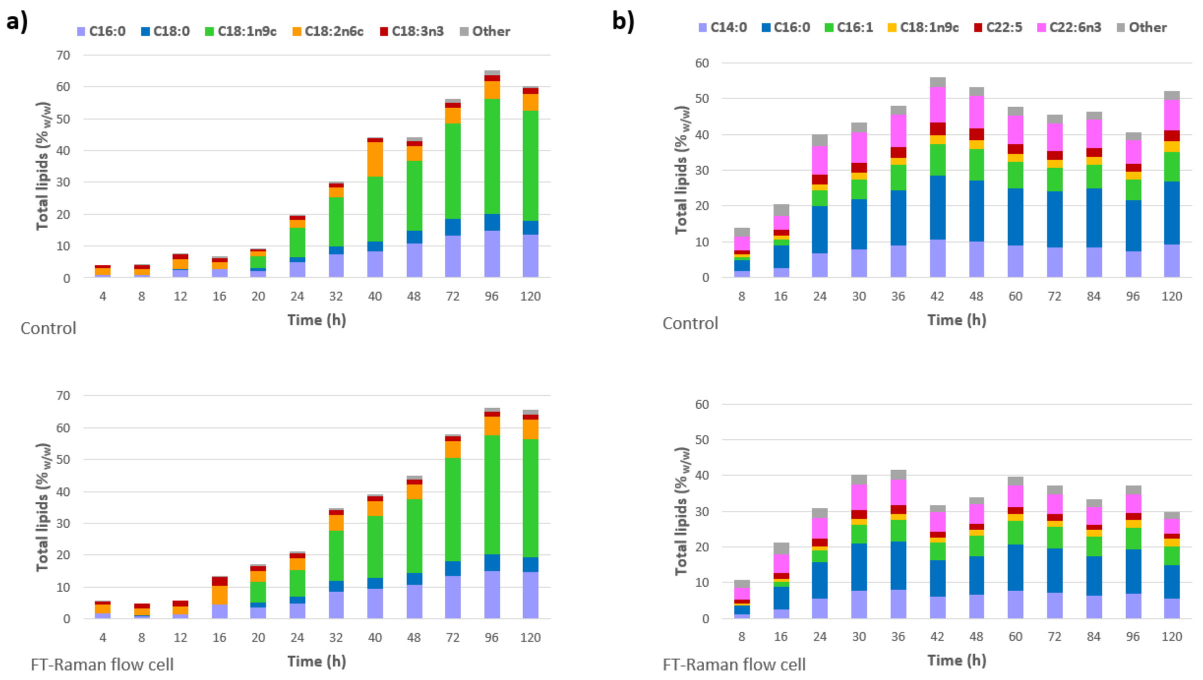

Figure S1. Relative lipid content ($‰_{w/w}$) and fatty acid composition for the control bioreactor (up) and the bioreactor with FT-Raman flow measurement cell in recirculatory loop (down) during (a) fermentation of glucose by *Rhodotorula*, and (b) fermentation of glycerol by *Schizochytrium*.

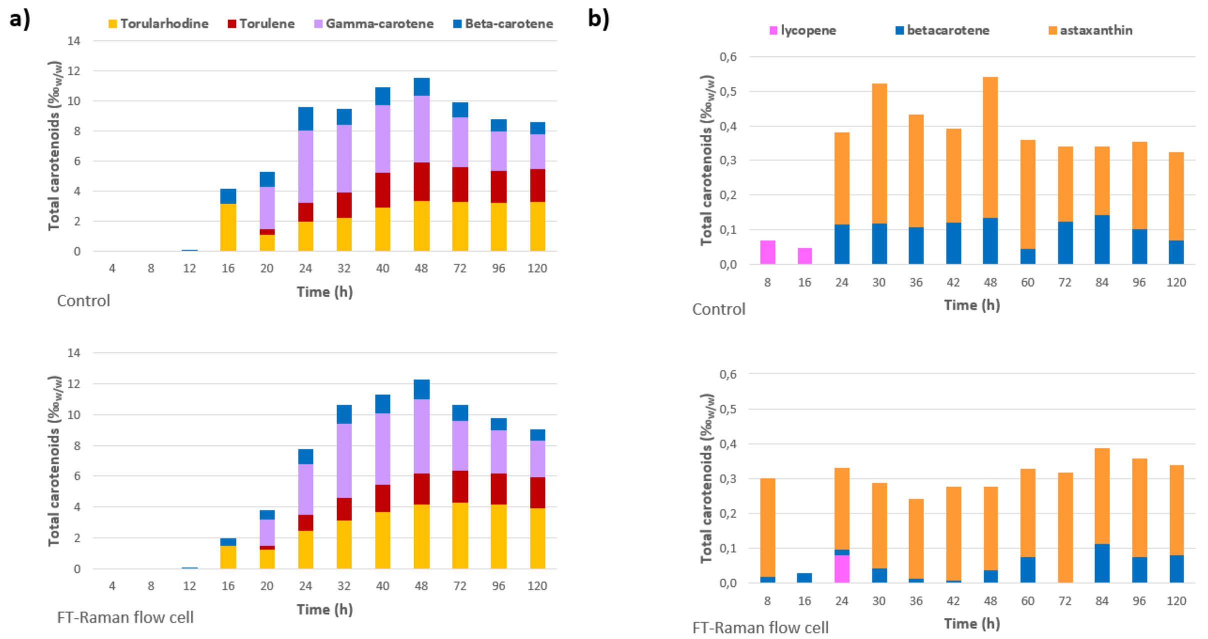

Figure S2. Relative carotenoid content ($‰_{w/w}$) and composition for the control bioreactor (up) and the bioreactor with FT-Raman flow measurement cell in recirculatory loop (down) during (**a**) fermentation of glucose by *Rhodotorula*, and (**b**) fermentation of glycerol by *Schizochytrium*.

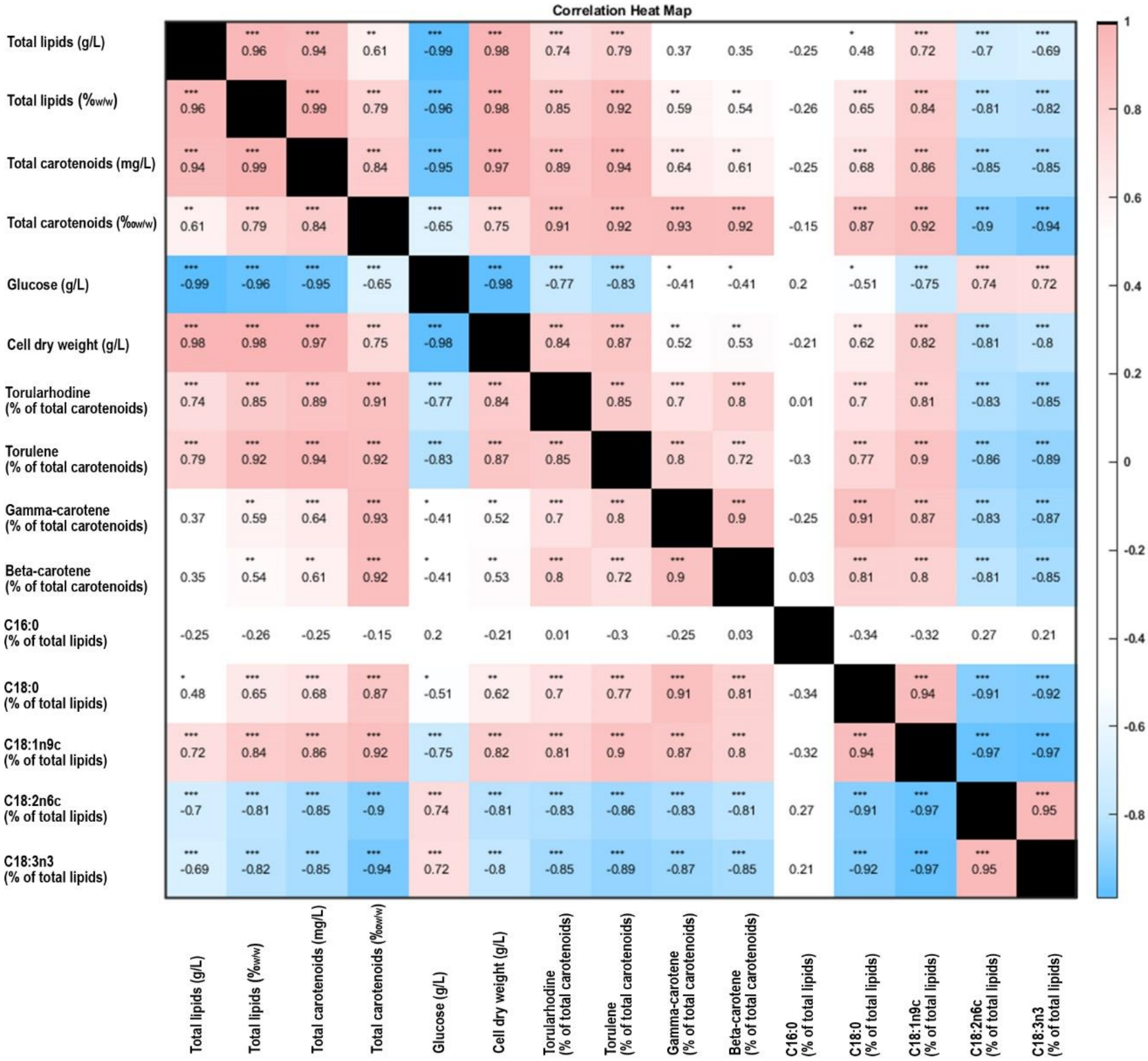

Figure S3. Correlation matrix of production parameters (relative content of individual carotenoids and FAME lipids) for fermentation of glucose by *Rhodotorula*, with Pearson correlation coefficients and statistical significance levels: *** for p<0.001; ** for p<0.01; * for p<0.05. The matrices include data from both bioreactors (control bioreactor and bioreactor with FT-Raman flow measurement cell).

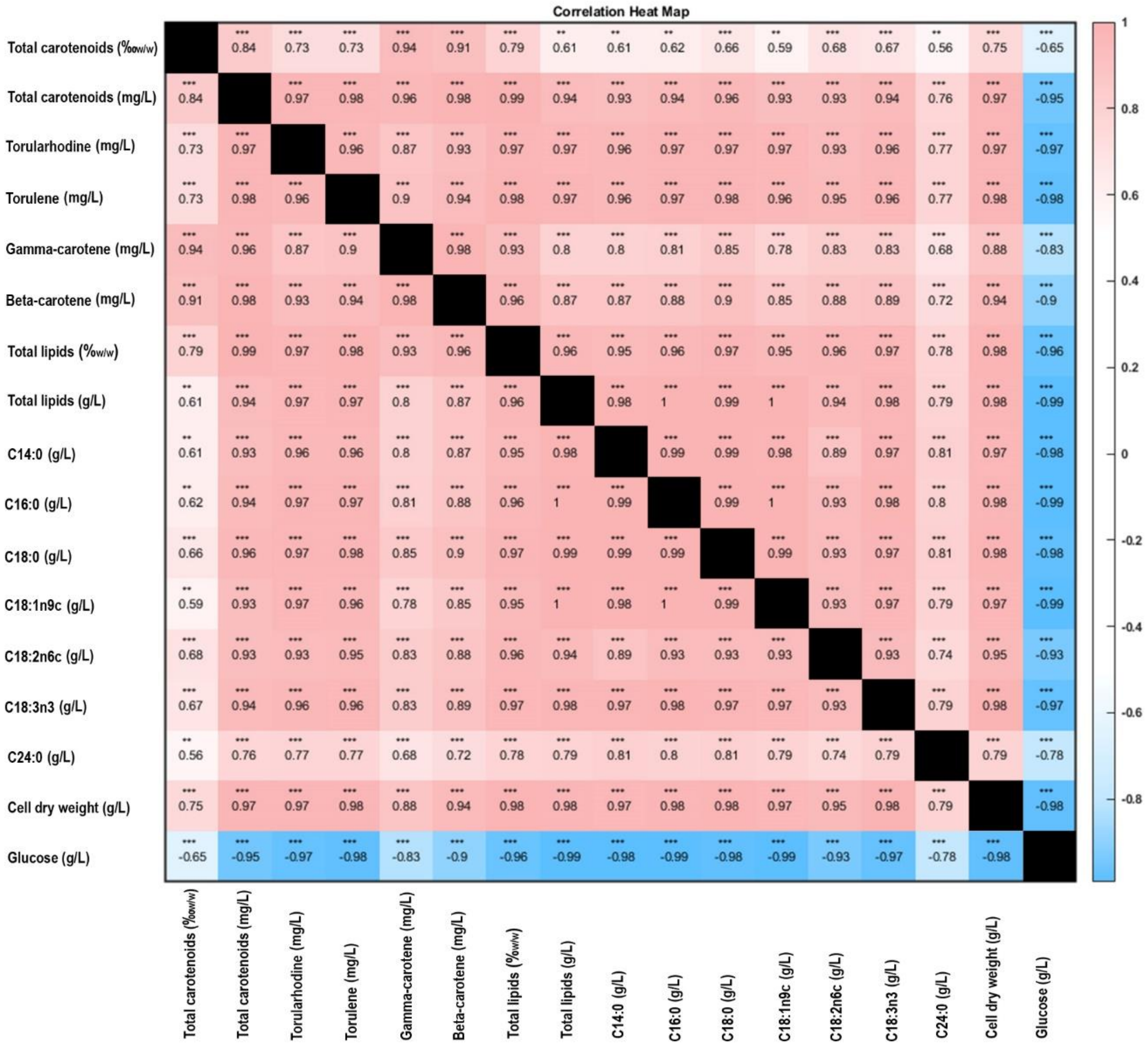

Figure S4. Correlation matrix of production parameters (concentrations of individual carotenoids and FAME lipids in mg/L and g/L, respectively) for fermentation of glucose by *Rhodotorula*, with Pearson correlation coefficients and statistical significance levels: *** for p<0.001; ** for p<0.01; * for p<0.05. The matrices include data from both bioreactors (control bioreactor and bioreactor with FT-Raman flow measurement cell).

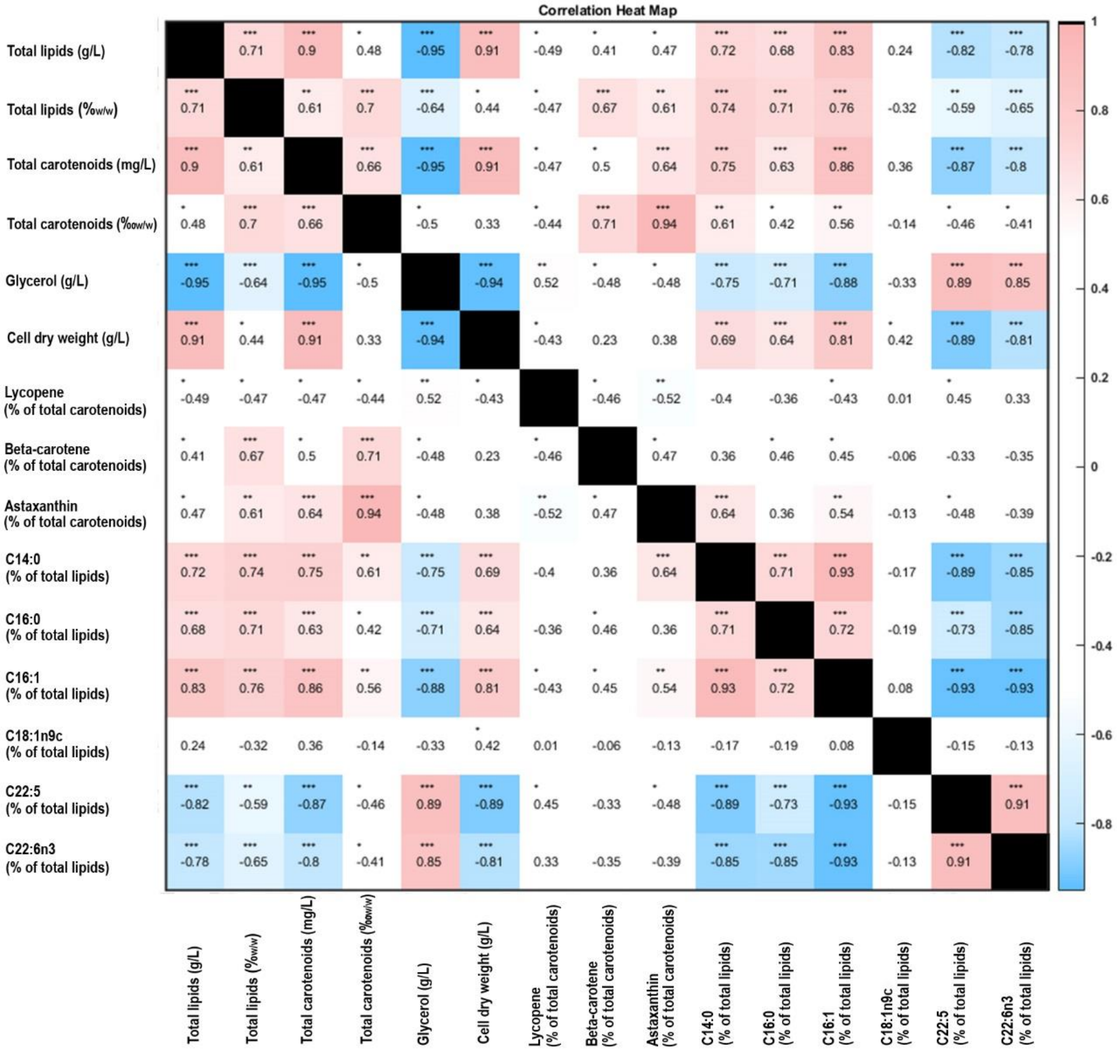

Figure S5. Correlation matrix of production parameters (relative content of individual carotenoids and FAME lipids) for fermentation of glycerol by *Schizochytrium*, with Pearson correlation coefficients and statistical significance levels: *** for p<0.001; ** for p<0.01; * for p<0.05. The matrices include data from both bioreactors (control bioreactor and bioreactor with FT-Raman flow measurement cell).

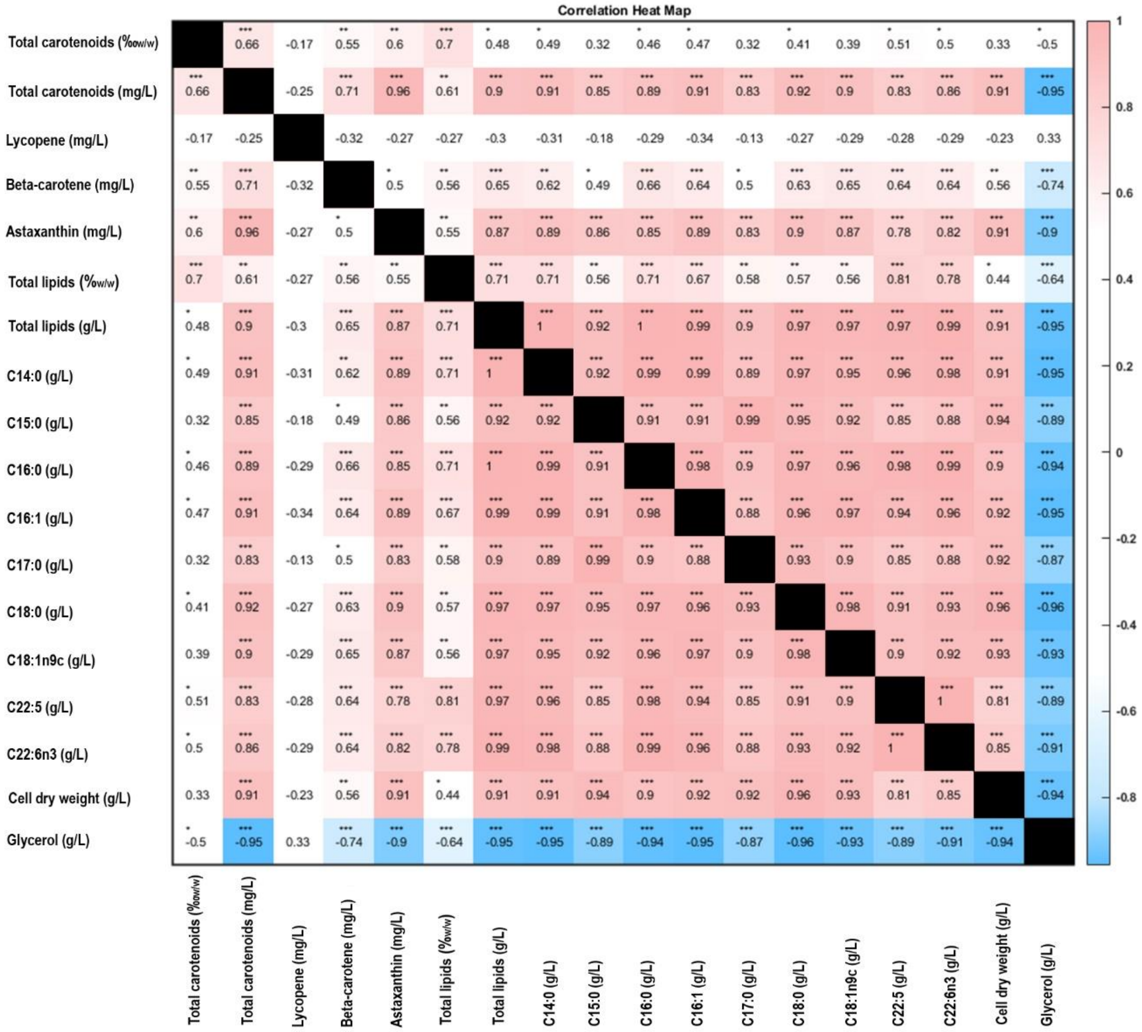

Figure S6. Correlation matrix of production parameters (concentrations of individual carotenoids and FAME lipids in mg/L and g/L, respectively) for fermentation of glycerol by *Schizochytrium*, with Pearson correlation coefficients and statistical significance levels: *** for p<0.001; ** for p<0.01; * for p<0.05. The matrices include data from both bioreactors (control bioreactor and bioreactor with FT-Raman flow measurement cell).

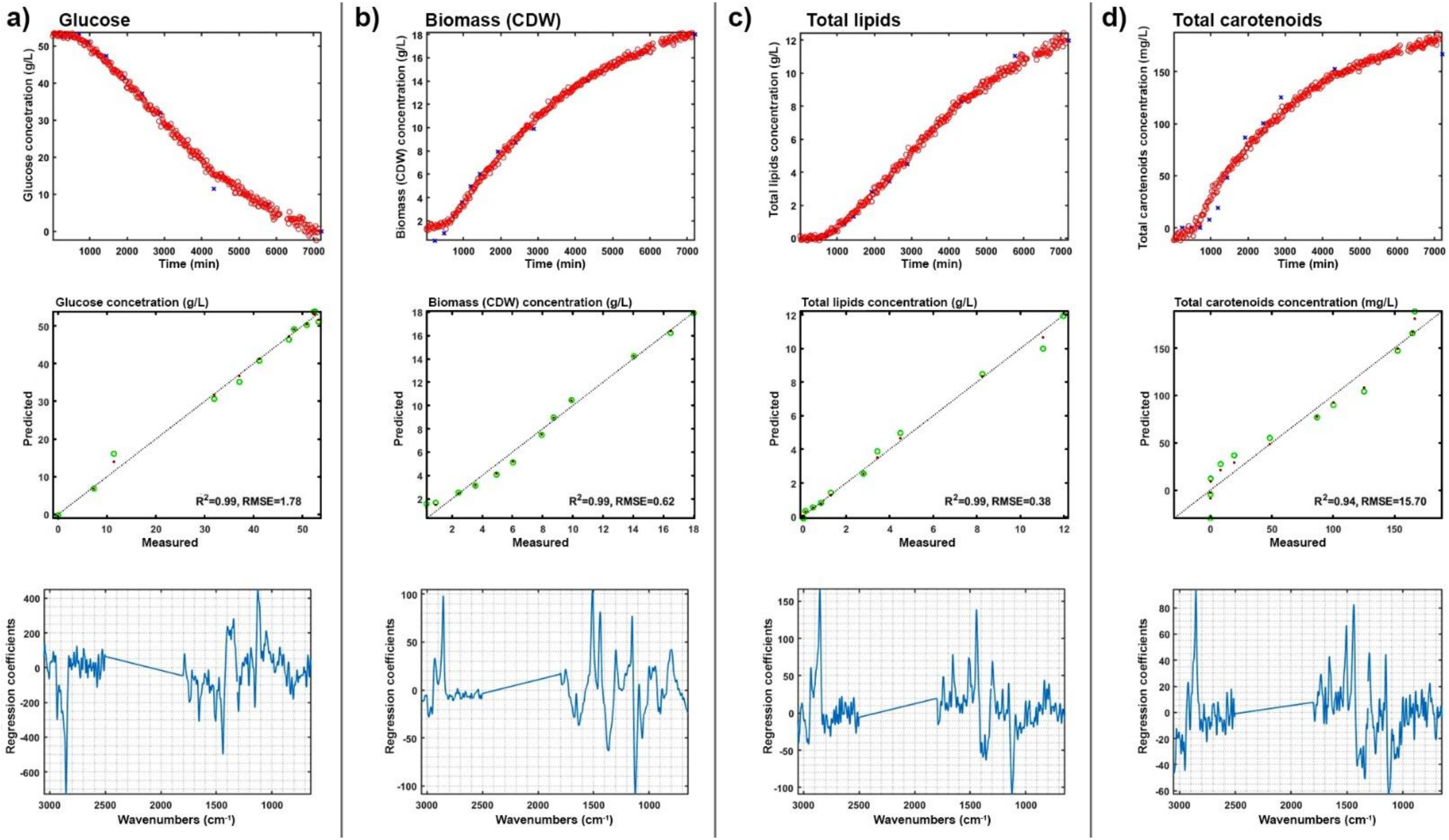

Figure S7. PLSR analysis of on-line spectroscopy data for fermentation of glucose by *Rhodotorula*. Concentration profiles (up) for glucose, biomass (CDW), total FAME lipids and total carotenoids (red ○), based on the best PLSR models established on regression of spectral data in selected timepoints on off-line reference data (blue ×). The corresponding PLSR models (middle) and regression coefficients (bottom).

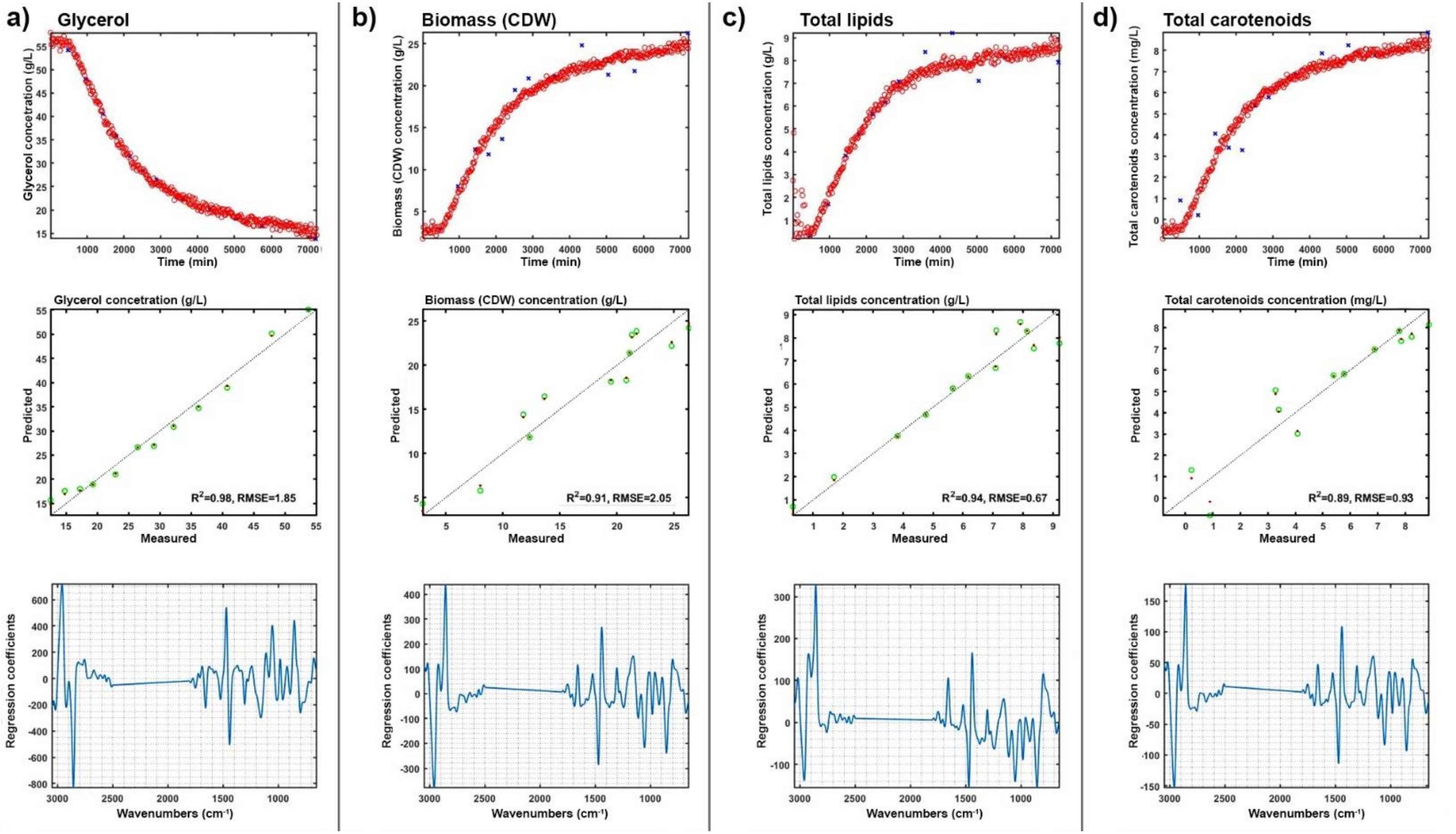

Figure S8. PLSR analysis of on-line spectroscopy data for fermentation of glycerol by *Schizochytrium*. Concentration profiles (up) for glycerol, biomass (CDW), total lipids and total carotenoids (red ○), based on the best PLSR models established on regression of spectral data in selected timepoints on off-line reference data (blue ×). The corresponding PLSR models (middle) and regression coefficients (bottom).

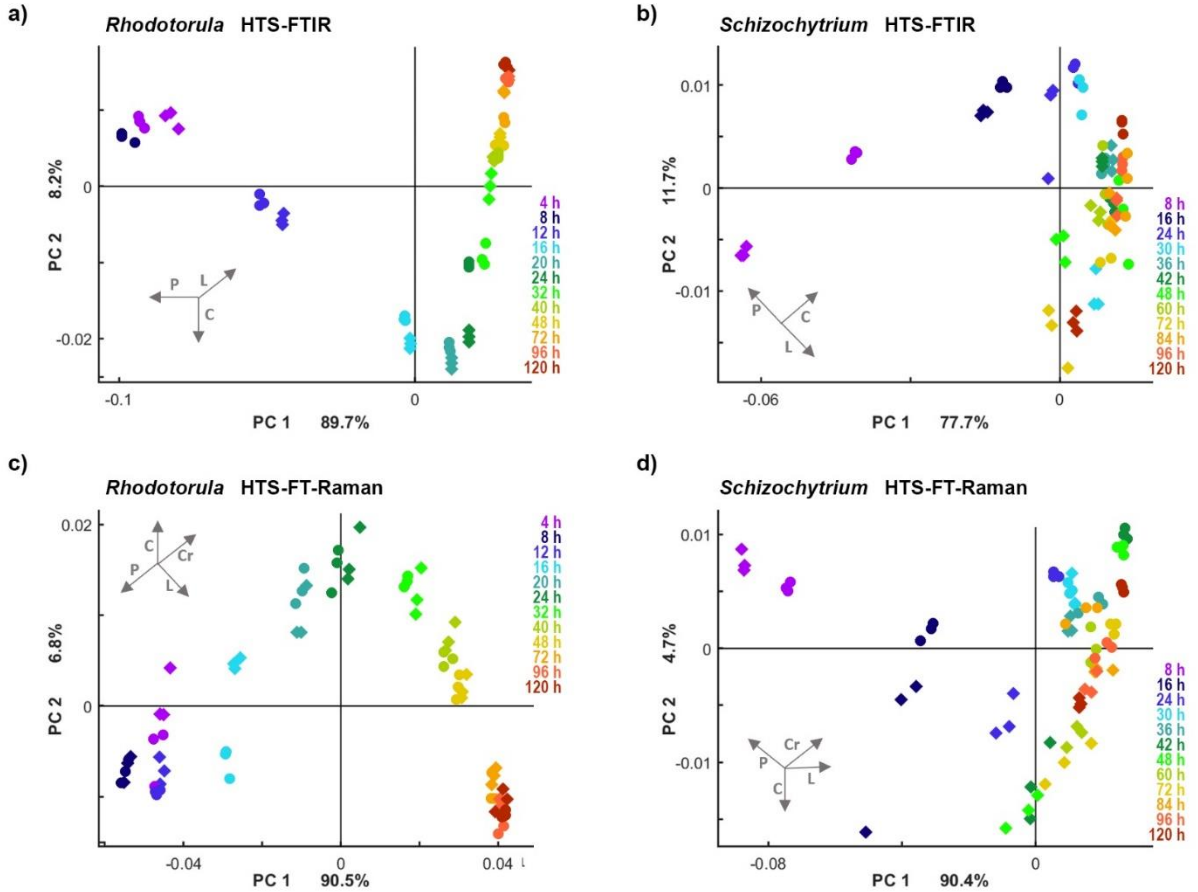

Figure S9. **Principal component analysis of at-line spectroscopy data**. PCA score plots of PC1 and PC2 of HTS-FTIR (up) and HTS-FT-Raman spectral datasets (down) of freeze-dried microbial biomass of bioprocess: (a and c) fermentation of glucose by *Rhodotorula*, and (b and d) fermentation of glycerol by *Schizochytrium*. (● - scores of the control bioreactor; ♦ - scores of the bioreactor with FT-Raman flow measurement cell in recirculatory loop). Vectors are approximating the increase in relative amount of the metabolites: lipids (L), proteins (P), cell wall carbohydrates (C), and carotenoids (Cr).

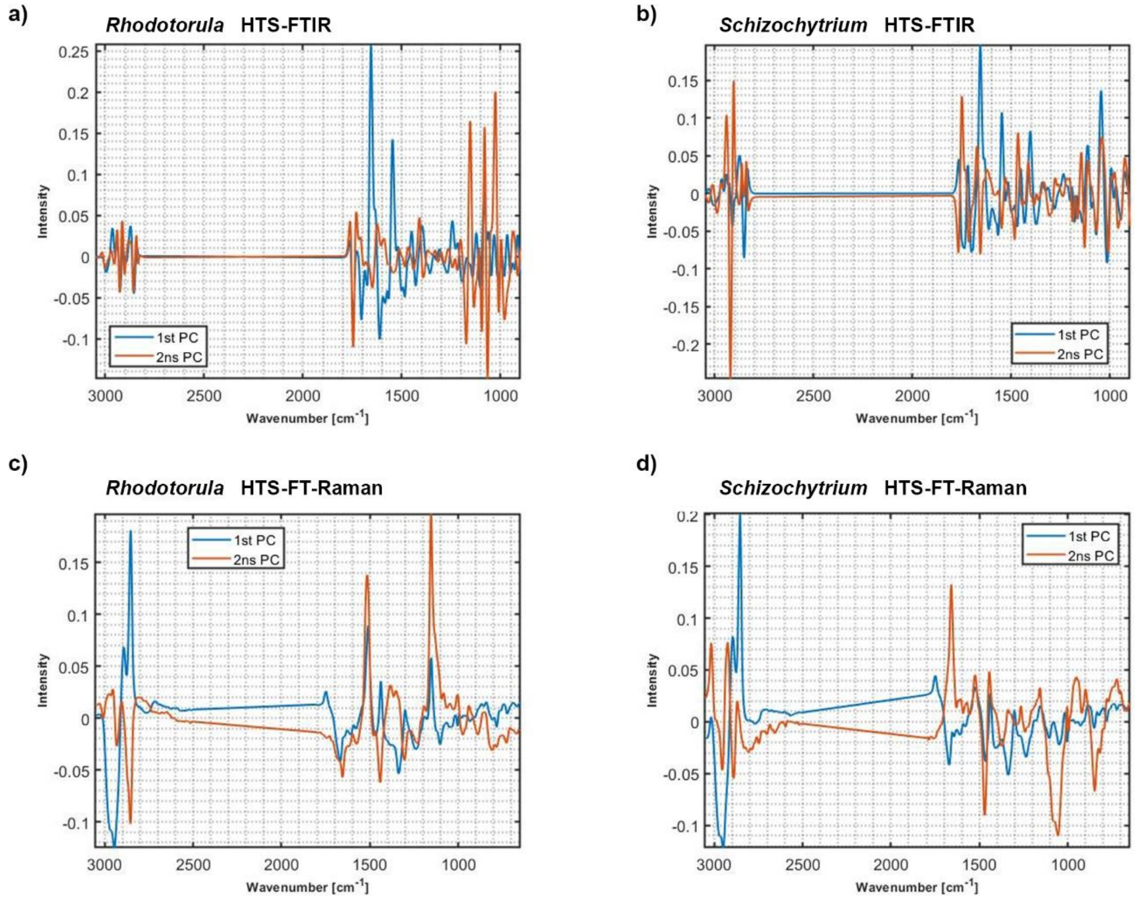

Figure S10. Principal component analysis of at-line spectroscopy data. PCA loadings plots on the first two principal components of HTS-FTIR (a, b) and HTS-FT-Raman spectral datasets (c, d) of freeze-dried microbial biomass of bioprocess: (a and c) fermentation of glucose by *Rhodotorula*, and (b and d) fermentation of glycerol by *Schizochytrium*.

Table S1. PLSR analysis of HTS offline (at-line) data. Coefficients of determination (R^2) and root mean square errors (RMSE) of prediction for determination of glucose/glycerol, cell dry weight, total FAME lipids, and total carotenoids, for the regression analyses with independent validation. The column names correspond to the datasets used for building the models. The models built on the data of the control bioreactor were tested (predicted) on the data of the bioreactor with FT-Raman flow measurement cell in recirculatory loop, and vice versa. The parameter ranges cover values for both bioreactors. R^2 and RMSE of cross validation of the PLSR models is presented in Tables 2 and 3.

**HTS-FT-Raman**	**Range**	**Control**		**Bypass loop**	
		**R^2_{test} (*Aopt*)**	**RMSE**	**R^2_{test} (*Aopt*)**	**RMSE**
Rhodotorula					
Glucose (g/L)	0.03-53.81	0.93 (2)	4.98 (9.2 %)	0.94 (2)	4.56 (8.5 %)
Cell dry weight (g/L)	0.32-18.60	0.96 (2)	1.21 (6.5 %)	0.96 (2)	1.20 (6.5 %)
Total lipids (% $_{w/w}$)	4.96-67.04	0.98 (3)	3.38 (5.0 %)	0.98 (2)	4.09 (6.1 %)
Total lipids (g/L)	0.02-12.18	0.93 (2)	1.15 (9.4 %)	0.94 (2)	1.14 (9.4 %)
Total carotenoids (‰$_{w/w}$)	0-12.62	0.92 (2)	1.55 (12.2 %)	0.84 (1)	1.89 (14.5 %)
Total carotenoids (mg/L)	0-166.51	0.99 (2)	8.93 (5.4 %)	0.98 (3)	9.93 (6.0 %)
Schizochytrium					
Glycerol (g/L)	12.53-54.17	0.94 (4)	11.79 (21.8 %)	0.94 (4)	8.25 (15.2 %)
Cell dry weight (g/L)	2.36-26.56	0.90 (2)	4.53 (17.1 %)	0.86 (4)	3.89 (14.6 %)
Total lipids (% $_{w/w}$)	10.87-56.13	0.60 (4)	7.64 (13.6 %)	0.89 (1)	9.35 (16.7 %)
Total lipids (g/L)	0.32-13.87	0.87 (2)	3.01 (21.7 %)	0.85 (4)	2.75 (19.8 %)
Total carotenoids (‰$_{w/w}$)	0.03-0.54	0.19 (1)	0.11 (20.4 %)	0.64 (1)	0.12 (22.2 %)
Total carotenoids (mg/L)	0.16-8.87	0.81 (3)	1.75 (19.7 %)	0.80 (5)	2.08 (23.4 %)
**HTS-FTIR**	**Range**	**Control**		**Bypass loop**	
		**R^2_{test} (*Aopt*)**	**RMSE**	**R^2_{test} (*Aopt*)**	**RMSE**
Rhodotorula					
Glucose (g/L)	0.03-53.81	0.81 (2)	8.50 (15.8 %)	0.89 (2)	6.56 (12.2 %)
Cell dry weight (g/L)	0.31-18.60	0.86 (2)	2.17 (11.7 %)	0.89 (2)	1.96 (10.5 %)
Total lipids (% $_{w/w}$)	4.96-67.04	0.92 (2)	7.11 (10.6 %)	0.93 (2)	6.08 (9.1 %)
Total lipids (g/L)	0.02-12.18	0.79 (2)	2.03 (16.7 %)	0.85 (2)	1.68 (13.8 %)
Total carotenoids (‰$_{w/w}$)	0-12.62	0.75 (1)	2.47 (19.6 %)	0.87 (2)	1.92 (15.2 %)
Total carotenoids (mg/L)	0-166.51	0.97 (2)	11.66 (7.0 %)	0.97 (2)	12.81 (7.7 %)
Schizochytrium					
Glycerol (g/L)	12.52-54.17	0.91 (3)	6.00 (11.1 %)	0.75 (5)	6.74 (12.4 %)
Cell dry weight (g/L)	2.36-26.56	0.92 (5)	3.59 (13.5 %)	0.89 (3)	2.29 (8.6 %)
Total lipids (% $_{w/w}$)	10.87-56.13	0.82 (1)	9.86 (17.6 %)	0.82 (1)	10.95 (19.5 %)
Total lipids (g/L)	0.32-13.87	0.88 (8)	2.37 (17.1 %)	0.75 (2)	2.42 (17.4 %)
Total carotenoids (‰$_{w/w}$)	0.03-0.54	0.06 (1)	0.14 (25.9 %)	0.53 (1)	0.14 (25.9 %)
Total carotenoids (mg/L)	0.16-8.87	0.82 (3)	1.29 (14,5 %)	0.83 (4)	1.33 (15.0 %)

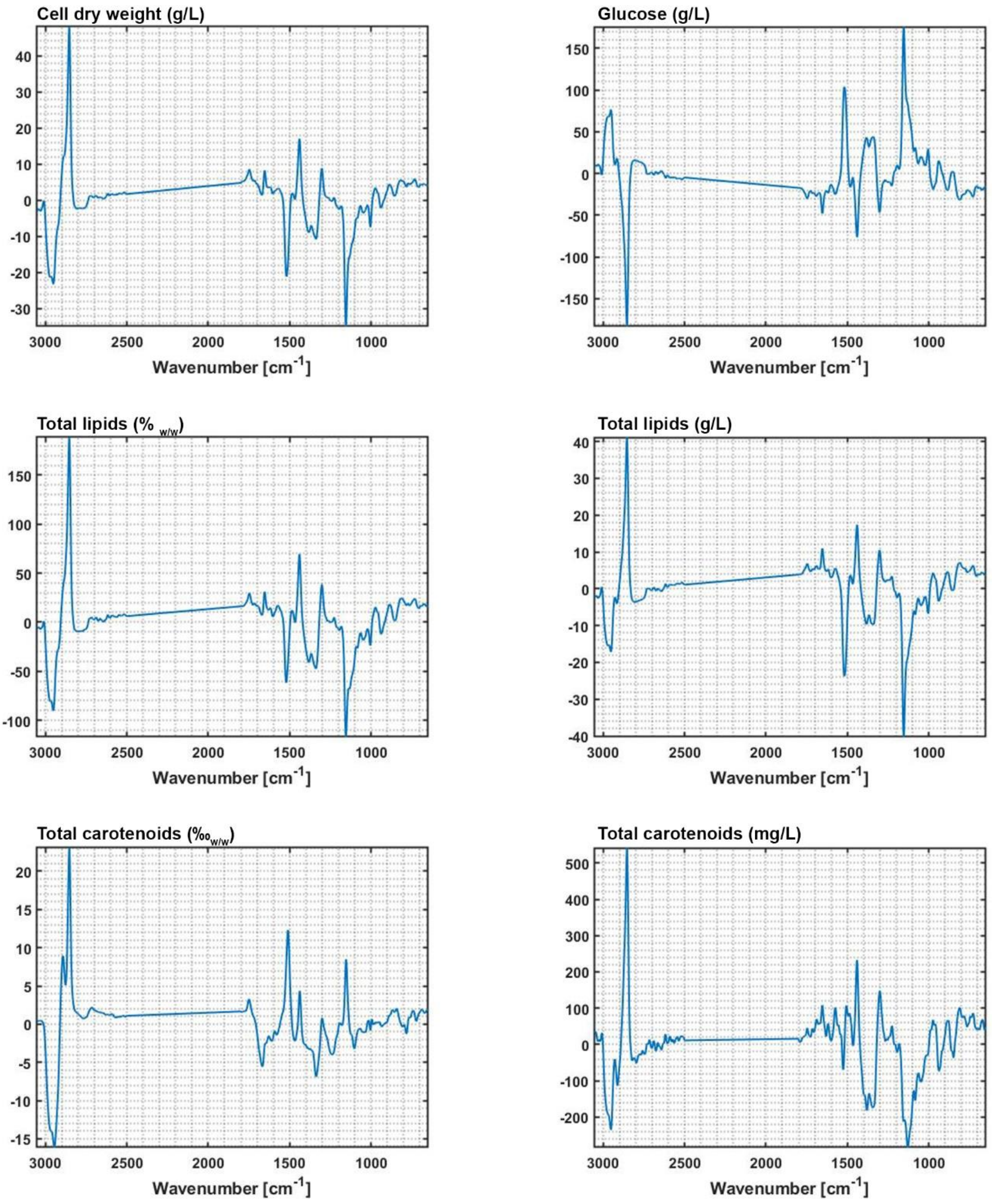

Figure S11. Regression coefficients of PLSR analyses of at-line HTS-FT-Raman spectral datasets for fermentation of glucose by *Rhodotorula*. The models were built on the data of the control bioreactor.

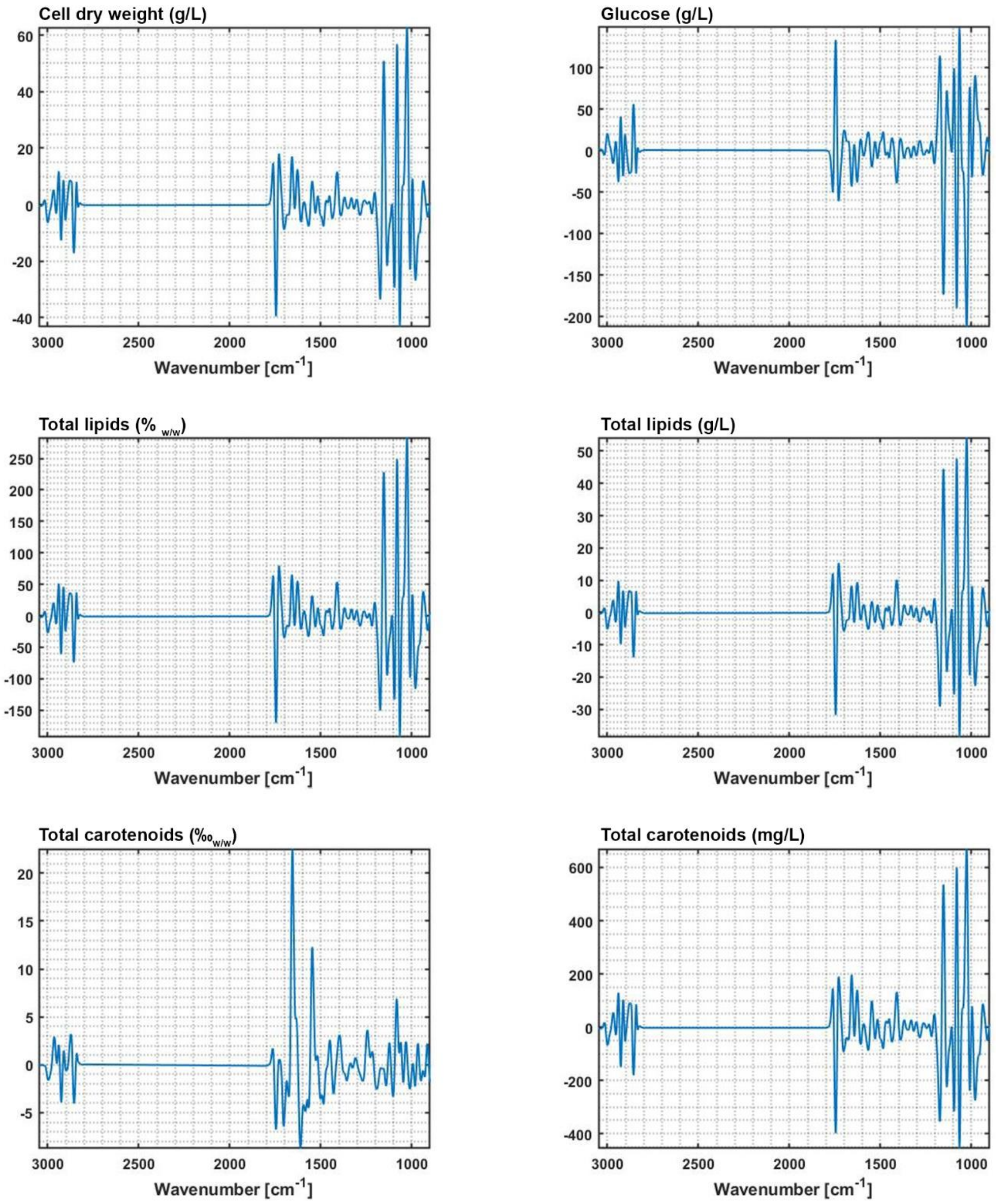

Figure S12. Regression coefficients of PLSR analyses of at-line HTS-FTIR spectral datasets for fermentation of glucose by *Rhodotorula*. The models were built on the data of the control bioreactor.

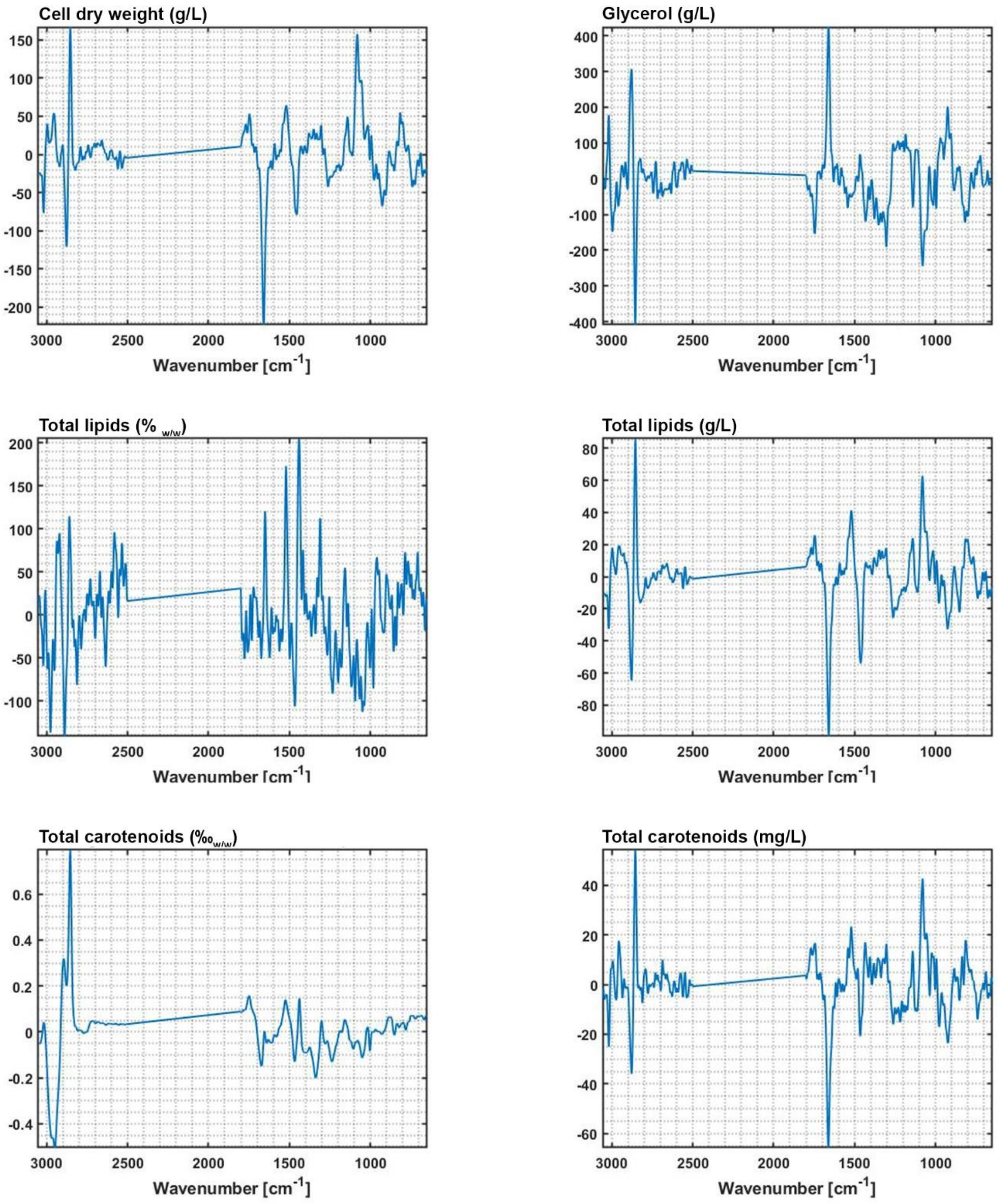

Figure S13. Regression coefficients of PLSR analyses of at-line HTS-FT-Raman spectral datasets for fermentation of glycerol by *Schizochytrium*. The models were built on the data of the control bioreactor.

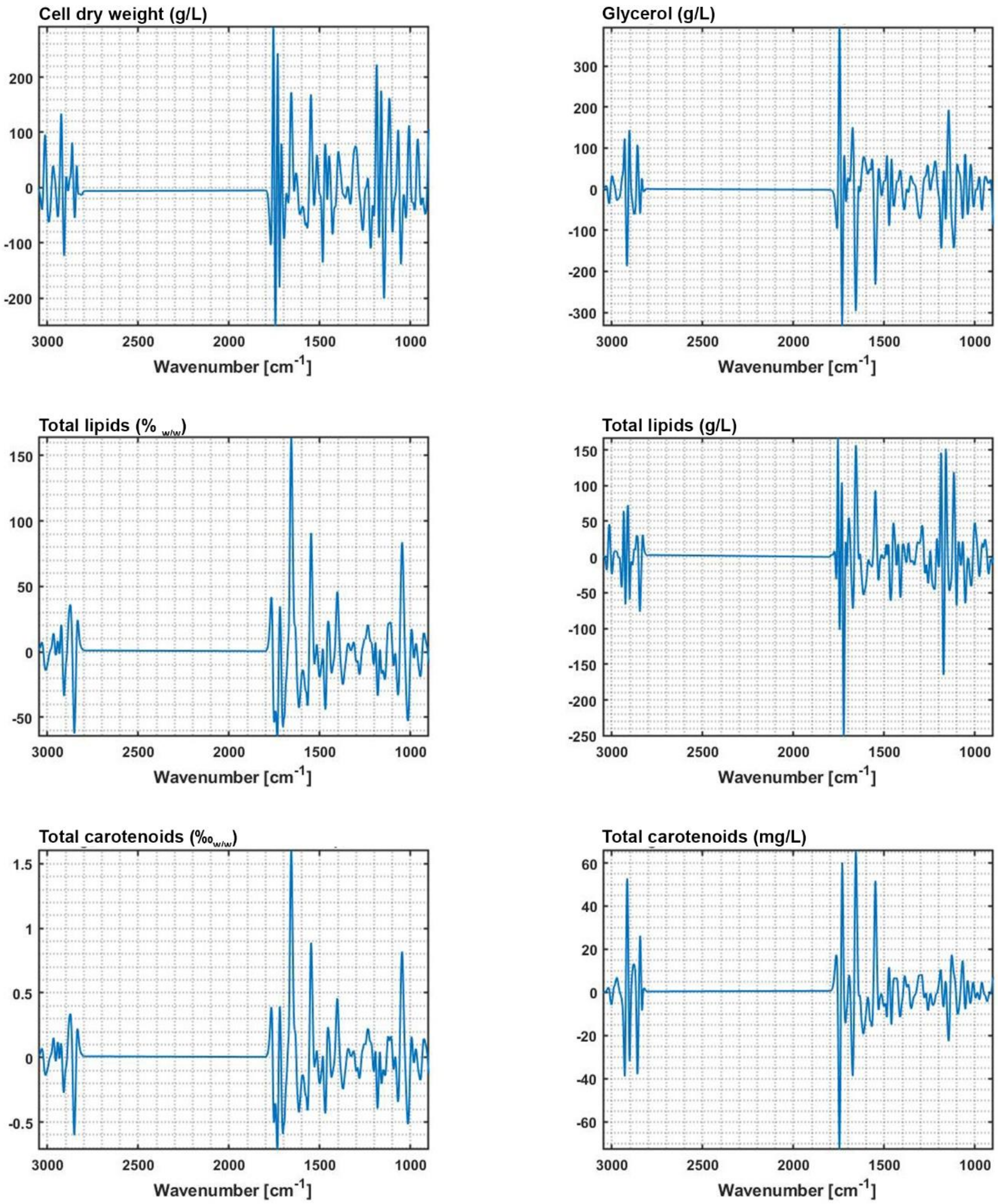

Figure S14. Regression coefficients of PLSR analyses of at-line HTS-FTIR spectral datasets for fermentation of glycerol by *Schizochytrium*. The models were built on the data of the control bioreactor.

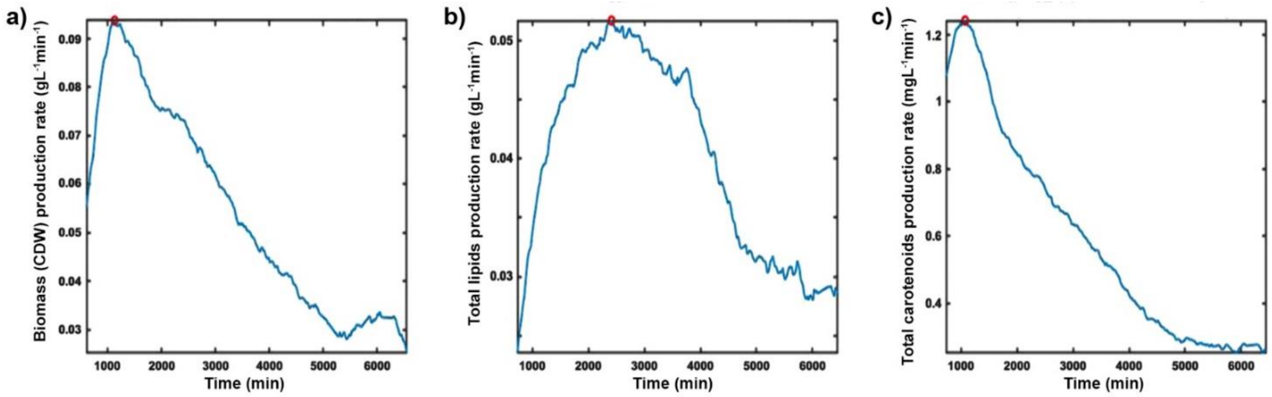

Figure S15. Production rates for fermentation of glucose by *Rhodotorula*. Production rates for: (a) biomass (CDW), (b) total lipids and (c) total carotenoids, based on the best PLSR models for online Raman measurements.
